# Titanium dioxide nanoparticles: a review of current toxicological data

**DOI:** 10.1186/1743-8977-10-15

**Published:** 2013-04-15

**Authors:** Hongbo Shi, Ruth Magaye, Vincent Castranova, Jinshun Zhao

**Affiliations:** 1 Public Health Department of Medical School, Zhejiang Provincial Key Laboratory of Pathological and Physiological Technology, Ningbo University, Ningbo, Zhejiang Province, 315211, P. R. China; 2 Pathology and Physiology Research Branch, Health Effects Laboratory Division, National Institute for Occupational Safety and Health, Morgantown, WV, 26505, USA

**Keywords:** Titanium dioxide, Nanoparticle, Toxicology, Toxicokinetics, Acute toxicity, Chronic toxicity, Genotoxicity, Reproductive toxicity, Carcinogenicity

## Abstract

Titanium dioxide (TiO_2_) nanoparticles (NPs) are manufactured worldwide in large quantities for use in a wide range of applications. TiO_2_ NPs possess different physicochemical properties compared to their fine particle (FP) analogs, which might alter their bioactivity. Most of the literature cited here has focused on the respiratory system, showing the importance of inhalation as the primary route for TiO_2_ NP exposure in the workplace. TiO_2_ NPs may translocate to systemic organs from the lung and gastrointestinal tract (GIT) although the rate of translocation appears low. There have also been studies focusing on other potential routes of human exposure. Oral exposure mainly occurs through food products containing TiO_2_ NP-additives. Most dermal exposure studies, whether *in vivo* or *in vitro*, report that TiO_2_ NPs do not penetrate the stratum corneum (SC). In the field of nanomedicine, intravenous injection can deliver TiO_2_ nanoparticulate carriers directly into the human body. Upon intravenous exposure, TiO_2_ NPs can induce pathological lesions of the liver, spleen, kidneys, and brain. We have also shown here that most of these effects may be due to the use of very high doses of TiO_2_ NPs. There is also an enormous lack of epidemiological data regarding TiO_2_ NPs in spite of its increased production and use. However, long-term inhalation studies in rats have reported lung tumors. This review summarizes the current knowledge on the toxicology of TiO_2_ NPs and points out areas where further information is needed.

## Introduction

With the development of nanotechnology, there has been a tremendous growth in the application of NPs for drug delivery systems, antibacterial materials, cosmetics, sunscreens, and electronics [1,2]. In October 2011 the European Union defined nanomaterials as a natural, incidental or manufactured material containing particles, in an unbound state or as an aggregate or agglomerate; where 50% or more of the particles exhibited, one or more external dimensions in the size range 1–100 nm [3]. Others have defined NPs as objects with at least one of their three dimensions in the range of 1–100 nm [4,5]. NPs generally possess dramatically different physicochemical properties compared to fine particles (FPs) of the same composition. The smaller size of NPs ensures that a large portion of atoms will be on the particle surface. Since surface properties, such as energy level, electronic structure, and reactivity are quite different from interior states, the bioactivity of NPs will likely differ from that of the fine size analogue.

Traditionally, TiO_2_ FPs have been considered as poorly soluble, low toxicity particles [6,7]. Due to this reason, they have been traditionally used as a “negative control” in many *in vitro* and *in vivo* particle toxicological studies [8]. However, this view was challenged after lung tumors developed in rats after two years of exposure to high concentrations of fine TiO_2_ particles [9]. The International Agency for Research on Cancer (IARC), therefore, has classified TiO_2_ as a Group 2B carcinogen (possibly carcinogenic to humans) [10]. However, the tumorigenic effect of fine TiO_2_ has been questioned and attributed to lung overload rather than specific carcinogenicity of fine TiO_2_[7]. In recent years, TiO_2_ NPs have been widely used in industrial and consumer products due to their stronger catalytic activity when compared to TiO_2_ FPs. This increase in catalytic activity has been attributed to their smaller sizes, which has allowed for larger surface area per unit mass. Concerns have been raised that these same properties of TiO_2_ NPs may present unique bioactivity and challenges to human health [11,12]. The rapid growth in the number of published studies confirms that there is a high level of interest concerning the safety of TiO_2_ NPs. Different animal models employing multiple exposure routes of administration, including inhalation, dermal exposure, intra-tracheal instillation, oral gavage, intragastric, intraperitoneal or intravenous injection have been intensively used in these studies. Studies have revealed that TiO_2_ NPs are more toxic than FPs [8,13,14]. Oberdorster *et al*. [15] reported that TiO_2_ NPs (21 nm) caused a greater pulmonary inflammatory response than TiO_2_ at same mass burden, with greater amounts of TiO_2_ NPs entering the alveolar interstitium in the lungs. Sager *et al*. [16] have reported similar results after intra-tracheal instillation of well-dispersed suspensions of TiO_2_ NPs (80/20 anatase/rutile; 21 nm, P-25) and TiO_2_ FPs (100% rutile; 1μm) in rats. On an equal mass burden, nano TiO_2_ was 40 fold more potent in inducing lung inflammation and damage at 1 and 42 days post-exposure than fine TiO_2_. However, respective potencies were not significantly different when dose was expressed on the basis of total surface area of particles delivered to the lung.

Wide application of TiO_2_ NPs confers substantial potential for human exposure and environmental release, which inevitably allows for a potential health risk to humans, livestock, and the eco-system [17]. This paper will focus mainly on current knowledge concerning the toxicology of TiO_2_ NPs. Studies done with mixtures of TiO_2_ NPs with other compounds and studies that have focused on aquatic ecosystems and the environment will not be discussed in this review. Even though the nanoparticle (NP) size has recently been defined as <100 nm, we have also included some studies that have defined particle sizes that are >100 nm as NPs. The molecular mechanisms of carcinogenesis will also be reviewed, to address health concerns regarding carcinogenesis due to particle exposure.

### Chemical and physical properties

Titanium (Ti), the ninth most abundant element in the earth's crust, is widely distributed. The average concentration of Ti in the earth's crust is 4400 mg/kg. Owing to its great affinity for oxygen and other elements, Ti does not exist in the metallic state in nature. The most common oxidation state of Ti is ^+^4, but ^+^3 and ^+^2 states also exist. Metallic Ti, TiO_2_, and TiCl_4_ are the compounds most widely used in industry. TiO_2_ (CAS-No. 13463-67-7), also known as titanium (IV) oxide, titanic acid anhydride, titania, titanic anhydride, or Ti white, is the naturally occurring oxide of Ti. TiO_2_ is a white noncombustible and odorless powder with a molecular weight of 79.9 g/mol, boiling point of 2972°C, melting point of 1843°C, and relative density of 4.26 g/cm^3^ at 25°C. TiO_2_ is a poorly soluble particulate that has been widely used as a white pigment. Anatase and rutile are two crystal structures of TiO_2,_ with anatase being more chemically reactive [18,19]. For example, Sayes *et al*. [19] reported that NPs (80/20; anatase/rutile, 3–5 nm; 100 μg/ml) generated 6 fold more reactive oxygen species (ROS) than rutile after UV irradiation. Indeed, anatase generates ROS when irradiated by UV light [19]. It has been suggested that TiO_2_ anatase has a greater toxic potential than TiO_2_ rutile [20,21]. However, anatase-generated ROS does not occur under ambient light conditions. TiO_2_ NPs are normally a mixture of anatase and rutile crystal forms. The principal parameters of particles affecting their physicochemical properties include shape, size, surface characteristics and inner structure. TiO_2_ FPs (the rutile form) are believed to be chemically inert. However, when the particles become progressively smaller, their surface areas, in turn, become progressively larger, and researchers have also expressed concerns about the harmful effects of TiO_2_ NPs on human health associated with the decreased size [22,23]. Surface modification such as coating, influences the activity of TiO_2_ NPs. For example, diminished cytotoxicity was observed when the surface of TiO_2_ NPs was modified by a *grafting*-*to* polymer technique combining catalytic chain transfer and thiol–ene click chemistry [24]. Another study confirmed the effect of surface coating on biological response endpoints of TiO_2_ NPs [25].

In conclusion, TiO_2_ NPs possess different physicochemical properties compared to TiO_2_ FPs. These properties likely influence bioactivity. Based on this fact, adverse health effects and environmental bio-safety of TiO_2_ NPs should be carefully evaluated even if TiO_2_ FPs have been demonstrated to have low toxicity. It is recommended that researchers carefully characterize the physicochemical properties of TiO_2_ NPs not only in the bulk form but also as delivered to the test system.

### Uses

TiO_2_ is a white pigment and because of its brightness and very high refractive index it is most widely used. Approximately four million tons of this pigment are consumed annually worldwide [26]. In addition, TiO_2_ accounts for 70% of the total production volume of pigments worldwide [27], and is in the top five NPs used in consumer products [28]. TiO_2_ can be used in paints, coatings, plastics, papers, inks, medicines, pharmaceuticals, food products, cosmetics, and toothpaste [29-31]. It can even be used as a pigment to whiten skim milk. TiO_2_ NPs are also used in sunscreens [32]. In addition, TiO_2_ has long been used as a component for articulating prosthetic implants, especially for the hip and knee [33,34]. These implants occasionally fail due to degradation of the materials in the implant or a chronic inflammatory response to the implant material [35].

Currently, TiO_2_ NPs are produced abundantly and used widely because of their high stability, anticorrosive and photocatalytic properties [4]. Some have attributed this increased catalytic activity to TiO_2_ NPs to their high surface area, while others attribute it to TiO_2_ NPs being predominantly anatase rather than rutile [18,19]. TiO_2_ NPs can be used in catalytic reactions, such as semiconductor photocatalysis, in the treatment of water contaminated with hazardous industrial by-products [36], and in nanocrystalline solar cells as a photoactive material [37]. Industrial utilization of the photocatalytic effect of TiO_2_ NPs has also found its way into other applications, especially for self-cleaning and anti-fogging purposes such as self-cleaning tiles, self-cleaning windows, self-cleaning textiles, and anti-fogging car mirrors [38]. In the field of nanomedicine, TiO_2_ NPs are under investigation as useful tools in advanced imaging and nanotherapeutics [37]. For example, TiO_2_ NPs are being evaluated as potential photosensitizers for use in photodynamic therapy (PDT) [39]. In addition, unique physical properties make TiO_2_ NPs ideal for use in various skin care products. Nano-preparations with TiO_2_ NPs are currently under investigation as novel treatments for acne vulgaris, recurrent condyloma accuminata, atopic dermatitis, hyperpigmented skin lesions, and other non-dermatologic diseases [40]. TiO_2_ NPs also show antibacterial properties under UV light irradiation [37,41].

### Exposure routes and limits

Ti occurs in tissues of normal animals but only in trace amounts [42]. There is no evidence of Ti being an essential element for human beings or animals. The Ti compound concentration in drinking water is generally low. A typical diet may contribute 300–400 μg/day. TiO_2_ particles are produced and used in varying particle size fractions including fine (approximately 0.1-2.5 μm) and nanosize (<0.1 μm, primary particles) [43]. Human exposure to TiO_2_ NPs may occur during both manufacturing and use. TiO_2_ NPs can be encountered as aerosols, suspensions or emulsions. The major routes of TiO_2_ NP exposure that have toxicological relevance in the workplace are inhalation and dermal exposure. It is reported that more than 150 items of “manufacturer-identified nanotechnology-based consumer products would have long term dermal contact. The most common nanomaterials found in consumer products for dermal application are TiO_2_ NPs [2]. TiO_2_ NPs are also widely used for toothpaste, food colorants and nutritional supplements. Therefore, oral exposure may occur during use of such products. In a recent study by Weir *et al*. [44] found that candies, sweets and chewing gums contained the highest amount of TiO_2_ in the scale of <100 nm. In nanomedicine, intravenous or subcutaneous injection of TiO_2_ nano particulate carriers is a unique way to deliver TiO_2_ NPs into the human body [45]. In cases where TiO_2_ NPs were embedded into products such as household paint, they have been shown to be less harmful, unless liberated by sanding [46].

The United States Food and Drug Administration (FDA) approved TiO_2_ as a food color additive with the stipulation that the additive was "not to exceed 1% by body weight (BW) ". TiO_2_ was also approved by the United States FDA as a “food contact substance” in food packaging [47]. Due to the differences in the physicochemical properties of TiO_2_ FPs and NPs exposure and toxicity information for TiO_2_ NPs is needed to develop exposure limits specific for TiO_2_ NPs.

The American Conference of Governmental Industrial Hygienists (ACGIH) has assigned TiO_2_ FPs (total dust) a threshold limit value (TLV) of 10 mg/m^3^ as a time weighted average (TWA) for a normal 8 h workday and a 40 h workweek [48]. Permissible exposure limit (PEL)-TWA of the Occupational Safety & Health Administration (OSHA) for TiO_2_ FPs is 15 mg/m^3^[49]. In November 2005, the United States National Institute for Occupational Safety and Health (NIOSH) proposed a recommended exposure limit (REL) for TiO_2_ NPs at 0.3 mg/m^3^, which was 10 times lower than the REL for TiO_2_ FPs [50]. In the “Risk Assessment of Manufactured Nanomaterials TiO_2_ Executive Summary” compiled by the New Energy and Industrial Technology Development Organization (NEDO) in Japan, the acceptable exposure concentration of TiO_2_ NPs was estimated to be 1.2 mg/m^3^ as a TWA for a 8 h workday and a 40 h workweek [51]. The no observed adverse effect level (NOAEL) for P25 TiO_2_ was extrapolated to be around 1.2 mg/m^3^ respirable dust as TWA in the case of a hypothetical 8-hour day, 5-day working week.

Worker exposure is possible during the handling process. However, a study showed that exposure during handling, transferring, bagging, mixing, and oven cleaning was well below the currently established limits [52]. Lee *et al*. [53] monitored the occupational exposure at workplaces in Korea that manufacture TiO_2_ NPs. Personal sampling, area monitoring, real-time monitoring, and dust monitoring were conducted at workplaces where the workers handled TiO_2_ NPs. The gravimetric concentrations of TiO_2_ NPs ranged from 0.10 to 4.99 mg/m^3^. The particle numbers concentration at the TiO_2_ NPs manufacturing workplaces ranged from 11,418 to 45,889 particles/cm^3^ with a size range of 15–710 nm. Occupational exposure to engineered nanomaterial oxides could be effectively reduced by proper local exhaust ventilation (LEV), filtration, containment, and good work practices [54].

In conclusion, the primary route of occupational exposure for TiO_2_ NPs is inhalation. Consumer inhalation is also possible during application of antimicrobial spray containing TiO_2_ NPs. Oral exposure may occur through food products which contain TiO_2_ NP-additives. Dermal contact may occur through applications of cosmetics and sunscreens. Intravenous injection of TiO_2_ NPs could occur in medical application. Further information is needed regarding airborne exposure levels of TiO_2_ NPs in the workplace and what processes are associated with high NP releases. It is recommended that workplace exposure assessment evaluate airborne mass particle concentrations, particle counts, and size distribution of TiO_2_ NPs. To date, the consumer or environmental exposure data for TiO_2_ NPs is inadequate. Therefore, it is recommended that exposure assessment be made throughout the life cycle of products containing TiO_2_ NPs.

### Toxicokinetics

Toxicokinetics is the description of the rate at which a substance (TiO_2_ NPs) enters the body through different exposure routes and its fate after entering the body. The level or concentration of TiO_2_ NPs in the body system depends on the rate (or kinetic) of absorption, distribution, metabolism, and excretion of TiO_2_ NPs. These processes may occur after exposure through inhalation, ingestion, dermal contact, and intraperitioneal or intravenous injection. The toxicokinetics of TiO_2_ NPs will be discussed in terms of the different kinetics.

#### Absorption

Following deposition of NPs at the initial site of exposure, absorption and translocation to systemic sites is a critical step in toxicokinetics. It is often defined as migration of the NP to distal organs. For instance, at what rate are TiO_2_ NPs absorbed through the GIT, the skin (dermal), pulmonary system, or other exposure sites, as with intravenous exposure, intra-peritoneal exposure, or subcutaneous exposure.

#### Gastrointestinal absorption

GIT may be an important absorption route for TiO_2_ NPs since drug carriers, food products, water and liquid beverages may contain TiO_2_ NPs [55,56]. In the field of nanomedicine, the GIT uptake of NPs has been the subject of recent efforts to develop effective carriers that enhance the oral uptake of drugs and vaccines [57]. TiO_2_ FPs (rutile; 500 nm; 12.5 mg/kg) have been shown to systemically translocate to other tissues from the rat GIT [58]. TiO_2_ particle uptake in GIT was proposed to take place *via* the peyers patches, due to the high presence of TiO_2_ FPs in the lymphoid tissues. TiO_2_ NPs have also been shown to be absorbed from the GIT (25, 80, and 155 nm; 5 g/kg BW; single oral dose; mice) [59]. TiO_2_ NPs may be absorbed through the GIT through the lymphoid tissues surrounding it. However, since the dose used in this study was high, the extent of absorption under relevant human exposures is in question.

#### Dermal absorption

Dermal absorption of TiO_2_ NPs is of interest because many consumer products, such as cosmetics and sunscreens may contain TiO_2_ NPs. The outer skin of human beings consists of a tough layer of SC that is difficult for inorganic particles to penetrate. Theoretically, only those materials with an appropriate octanol/water partition coefficient and low molecular weight (<ca. 500) should penetrate the intact human skin through the SC. Therefore, it is unlikely that inorganic particles would penetrate the intact skin under normal conditions [60]. It is worth noting that although cosmetics and sunscreens containing TiO_2_ are normally used on intact skin. Slight injuries to skin can occur under certain circumstances, such as physical force or sunburn. Thus, skin penetration studies of TiO_2_ particles are usually investigated *in vivo* and *in vitro* with both intact skin and stripped skin which mimics an injured skin [60]. Several studies have investigated dermal penetration by TiO_2_ NPs. Some of these studies [61-65] concluded that TiO_2_ NPs did not penetrate the intact human skin. Senzui *et al*. [60] investigated the skin penetration of four types of rutile TiO_2_ particles (35 nm non-coated, 35 nm coated, 100 nm almina and silicon coated, and 250 nm non-coated) in intact or stripped skin of Yucatan micropigs *in vitro* (2 μl suspension, 1cm^2^ skin area). Results demonstrated that TiO_2_ particles did not penetrate viable skin, even though the particle size was less than 100 nm and the SC was damaged. Further observation with scanning electron microscopy (SEM) showed that although some TiO_2_ particles had lodged into vacant hair follicles, it did not penetrate the dermis or viable epidermis. Similar results were obtained previously by Lademann *et al*. [66], showing no penetration into live tissue. Tape stripping with adhesive tape is a widely accepted method to examine the localization and distribution of substances within the SC [67]. Tan *et al*. [68] investigated a sunscreen containing 8% TiO_2_ NPs (10–50 nm) applied twice a day for 2–6 weeks on the skin of human volunteers (age range 59–82 yr) and evaluated the epidermal penetration of TiO_2_ NPs into the epidermis using tape stripping. They found that levels of TiO_2_ NPs in the epidermis and dermis of subjects who applied TiO_2_ NPs to their skin were higher than the levels of TiO_2_ NPs found in controls. However, they pointed out that a larger sample size would be necessary to establish if this difference was significant. It is also worth noting that the morphology of the SC differs among different age groups, which also influences the results. Bennat and Müller-Goymann [69] showed that TiO_2_ NPs penetrated hairy skin when applied as an oil-in-water emulsion. They evaluated skin penetration of TiO_2_ NPs (20 nm, formulations with 5% TiO_2_ NPs) applied to human skin either as an aqueous suspension or as oil-in-water emulsion using the tape stripping method. The results suggest that TiO_2_ NPs maybe able to penetrate the surface through hair follicles or pores. However, no details were given regarding the fate of the particles that did penetrate. TiO_2_ NPs were also found to have no skin carcinogenesis promoting effects due to lack of penetration through the epidermis [70,71] (details given in the section on carcinogenecity). Another study utilizing the time of flight secondary ion mass spectrometry (TOF-SIMS) showed the presence of silica coated TiO_2_ NPs (rutile; 14–16 nm) within epidermis and superficial dermis [72]. They concluded that ultraviolet-B (UVB) damaged skin (pigs; UVB sunburn; 250 μl of each sunscreen formulation) slightly enhanced TiO_2_ NPs penetration in sunscreen formulations but they did not detect transdermal absorption.

Similar results were obtained in an *in vitro* study [73]. In this study the cutaneous penetration and localization of TiO_2_ NPs (≥20 nm primary size; 24 h sunscreen application), included in a sunscreen was evaluated in intact, damaged, irradiated, and damaged/irradiated pig skin. Quantitative analysis was done using an inductively coupled plasma-mass spectrometry, qualitative analysis done using transmission electron microscopy (TEM) and elemental identity of the NPs was evaluated by TEM-coupled Energy Dispersive X-ray (TEM-EDX) analysis. In intact and damaged/irradiated skins, 102.35±4.20% and 102.84±5.67% of the Ti was deposited, respectively, at the surface and SC, whereas only 0.19±0.15% and 0.39±0.39% were found in the viable epidermis and dermis. No Ti was detected in the receptor fluid. TEM-EDX analysis confirmed the presence of TiO_2_ NPs at the SC surface, as already characterized in the sunscreen formulation. They concluded that TiO_2_ NPs included in a sunscreen remain in the uppermost layers of the SC, in intact skin, compromised skin, or skin exposed to simulated solar radiation. Filipe *et al*. [74] also noted that in normal human skin TiO_2_ NPs were unlikely to penetrate the SC towards the underlying keratinocytes (coated 20 nm; 2 and 48 h).

One study found that TiO_2_ NPs could possibly penetrate the SC depending on the particle coating and skin with or without hair. However, their claims may be questioned, due to lack of data on systemic evaluation of the NPs that did penetrate the SC. Apart from this, it should be noted that most other studies reported that TiO_2_ NPs did not penetrate into live tissue from the deposition sites. Therefore, it can be concluded that TiO_2_ NPs are not systemically available to a significant extant after dermal exposure.

#### Pulmonary absorption

The pulmonary system consists of the upper respiratory tract (nose and nasal passages, paranasal sinuses and pharynx) and the lower respiratory tract (larynx, trachea, bronchi and lungs). Here we include studies done through inhalation, intratracheal instillation and intra-nasal (oro-pharyngeal) exposure.

##### Inhalation exposure

Inhalation is one of the major routes for TiO_2_ NPs to gain entry into the human body especially in occupational settings. Numerous studies have used inhalation as the exposure route to determine the toxicokinetics and cyto- or genotoxicity of TiO_2_ NPs. The limit for FPs in the air is 50 μg/m^3^ for an average human of 70 kg [75].

**Figure 1:**
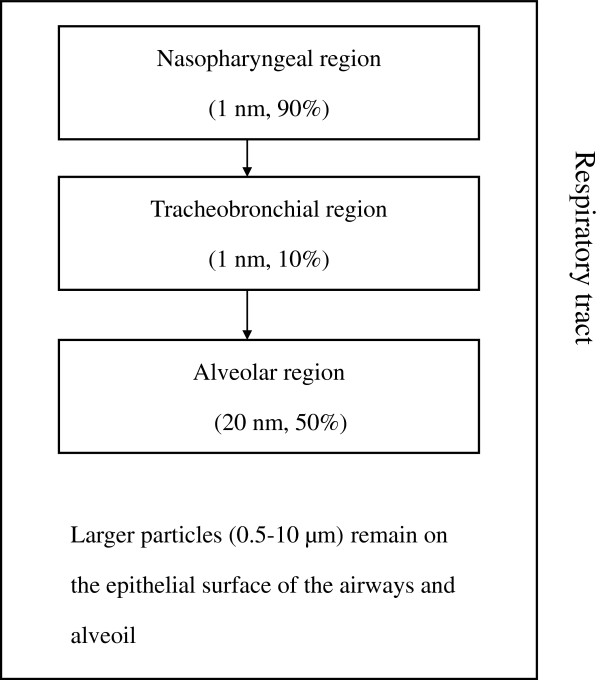
**Particulate distribution of TiO**_**2 **_**particles by size through different regions of the respiratory tract.** This diagram was derived from an explanation of particulate distribution after inhalation given by Simko and Mattsson [75]. Arrows represent downward movement of particles through the respiratory tract. Most particles in the size range of 1–5 nm are distributed throughout the three regions. 1 nm and 20 nm particles are mostly distributed in the nasopharyngeal region and alveolar regions respectively. 0.5-10 μm particles remain on the epithelial surface of the airways and alveoli.

Figure 1 shows the particulate distribution of TiO_2_ particles by size through the different regions of the respiratory tract after inhalation.

Human data related to absorption through inhalation of TiO_2_ NPs are currently not available. However, there are quantitative data available from rodent studies [76]. Muhlfeld *et al*.[77] suggested that a small fraction of TiO_2_ NPs (20 nm; 1 and 24 h; dose ranges differed according to compartment size) are transported from the airway lumen of adult male WKY/NCrl BR rats to the interstitial tissue and subsequently released into the systemic circulation.

##### Intratracheal instillation

Intratracheal instillation is a technique where single or repeated doses of precise volumes of particulate suspensions are administered directly to the lungs. It should be noted that there may be significant differences in distribution, clearance, and retention of materials administered by intratracheal instillation, especially at high bolus doses, compared to low dose rate inhalation. Although, inhalation studies are considered to be the gold standard, data from intratracheal instillation studies can be used for hazard assessment [78]. Sager *et al*. [16] reported that a significant portion of deposited TiO_2_ NPs (21 nm) migrated to the interstitial space by 42 days after intratracheal instillation in rats. TiO_2_ NPs migrated to the alveolar interstitium to a significantly greater extent than TiO_2_ FPs after either inhalation exposure [15] or intratracheal instillation [16]. Another study found that at 28 days after instillation, a small fraction of pulmonary TiO_2_ NPs were able to access the blood circulation and reach extrapulmonary tissues such as liver and kidneys [79].

##### Intranasal exposure

Breathing is mostly done through the nose, and is termed nasal breathing. The nasal cavity has two segments, the respiratory segment and the olfactory segment. The respiratory segment is lined with ciliated pseudostratified columnar epithelium, it has a very vascularized lamina propria allowing the venous plexuses of the conchal mucosa to engorge with blood, restricting airflow and causing air to be directed to the other side of the nose. The olfactory segment is lined with the olfactory epithelium, which contains receptors for the sense of smell. Olfactory mucosal cell types include bipolar neurons, supporting (sustentacular) cells, basal cells, and Bowman's glands. The axons of the bipolar neurons form the olfactory nerve (cranial nerve I) and enter the brain through the cribiform plate. Studies by Wang *et al*. [80,81] on murine brain reported that intra-nasally instilled TiO_2_ NPs (80 nm rutile, 155 nm anatase; 500 μg/ml; 2, 10, 20, and 30 days) can be taken up by sensory nerves and translocate to the brain.

Even though the inhalation, intratracheal instillation and intranasal studies in regards to pulmonary absorption are few they suggest that TiO_2_ NPs can translocate from the lung into the circulatory system to systemic tissue and from the nasal cavity into sensory nerves to the nervous system. Available data suggest that the rate of NP migration to the circulatory system is low.

#### Distribution

After the initial absorption of TiO_2_ NPs, the systemic circulation can distribute the particles to all organs and tissues in the body. After TiO_2_ NPs reach the systemic circulation, these particles potentially interact with plasma–proteins [82], coagulation factors, platelets and red or white blood cells. The binding to plasma components may have a substantial effect on the distribution, metabolism, and excretion of the NPs [55]. Binding to plasma components might neutralize or mask the adverse effects of TiO_2_ NPs in the systemic circulation. Therefore the biokinetics of the engineered NPs is also dependent on the local corona environment [83]. They might also contribute to disturbances in the corona environment as noted by Mikkelsen *et al*. [84]. In this study repeated exposure to TiO_2_ NPs (12–21.6 nm; 0.5 mg/kg BW) was associated with modest plaque progression in ApoE (−/−) mice. TiO_2_ NPs (20–30 nm, anatase 99.9%; 5 μg/ml) were also able to penetrate human red blood cells [85]. This cellular uptake of TiO_2_ NPs might not involve endocytosis, since erythrocytes do not have phagocytic receptors. These results imply that TiO_2_ NPs might be able to cross the cell membrane by processes other than phagocytosis and endocytosis. Diffusion or adhesive interactions may also play certain roles in this cellular uptake of TiO_2_ NPs [55,86]. Wick *et al*. [87] used the *ex vivo* human placental perfusion model to determine whether NPs can cross the blood-placenta barrier and whether this process is size dependent. Fluroescent polystyrene particles were used as a model NP. They found that fluorescent polystyrene particles with diameter up to 240 nm were taken up by the placenta. Earlier studies by Shimizu *et al*. [88] and Takeda *et al*. [89] had shown that subcutaneous exposure of pregnant mice to TiO_2_ NPs affected gene expression and genital and cranial nerve systems of the offspring. In addition, a study using inhalation exposure has shown that TiO_2_ NPs can also penetrate the blood placenta barrier [88-90].

Translocation refers to the way particles move from the site of absorption to other parts of the body. In humans it may occur in the alveolar region where the air-blood-barrier is approximately 2 μm thick. Geiser and Kreyling [91] in their review reported that NPs including TiO_2_ NPs in the size range of 5–100 nm could be translocated across the air-blood-barrier. When TiO_2_ NPs are translocated into the blood, generally they may be retained in the liver and lymphatic system, distributed to other organs and tissues, or eliminated out of the body. A 3-week inhalation study using nano- and fine TiO_2_ particles with 3, 28, and 90 days recovery time was performed in female Wistar rats [92]. This study observed that particles were mainly found in alveolar macrophages and, to a lesser extent, in type-I pneumocytes, and this was quantified using the relative deposition index (RDI). Particle-laden cells were rarely observed inside capillaries. They concluded that there was minimal translocation of particles into the blood stream.

The interactions between TiO_2_ NPs and alveolar macrophages and their associated pro-inflammatory effects in relation to particle size and physico-chemical properties was investigated *in vitro* by Scherbart *et al*. [93]. NR8383 rat lung alveolar macrophages were treated (10, 20, 40, and 80 μg/cm^2^; 1, 4, and 24 h) with TiO_2_ NPs (25 nm; 80/20; anatase/rutile), and FPs (250 nm). Alveolar macrophages rapidly took up both TiO_2_ NPs and FPs. Uptake inhibition experiments with cytochalasin D, chlorpromazine and a Fcγ receptor II (FcγRII) antibody revealed that the endocytosis of TiO_2_ FPs by the macrophages involved actin-dependent phagocytosis and macropinocytosis as well as clathrin-coated pit formation, whereas the uptake of TiO_2_ NPs was dominated by FcγIIR antibody. They suggested that the contrasting alveolar macrophage responses to TiO_2_ NPs and FPs relate to differences in the involvement of specific uptake mechanisms.

Ferin *et al*. [94] monitored pulmonary retention of TiO_2_ FPs and NPs in rats after a single intratracheal instillation or 12 week inhalation of different sizes of TiO_2_ particles (12, 21, 230, and 250 nm). They found that migration of particles to the interstitium appeared to be related to the particle size, the delivered dose, and the delivered dose rate. In addition, both acute instillation and sub-chronic inhalation studies showed that TiO_2_ NPs (20 nm) at equivalent masses access the pulmonary interstitium to a larger extent than TiO_2_ FPs (250 nm). A tracheal explants system study reported that TiO_2_ NPs (21 nm; 5 mg/ml; 1 h) enter the epithelium faster and are translocated in greater proportion to the subepithelial space compared with TiO_2_ FPs (0.12 μm) [95]. Li *et al*. [79] investigated the distribution and effects of TiO_2_ NPs (3 nm; 13.2 mg/kg; once a week for 4 weeks) in mice. They suggested that TiO_2_ NPs might pass through the blood–brain barrier.

Others have found that intra-nasally instilled TiO_2_ NPs could be translocated into the central nervous system *via* the olfactory nerves and cause potential brain lesions with the hippocampus being the main target [80]. These effects were mainly caused by the 155 nm anatase TiO_2_ particle, which they also defined as a NP. The same research group reported similar findings in another study (intranasal instillation; 80 nm rutile, 155 nm anatase; 30 days) [81]. The influence of intra-nasally instilled TiO_2_ NPs (25 nm, 80 nm and 155 nm; every other day in a dose of 50 mg/kg BW) on monoaminergic neurotransmitters (norepinephrine (NE), dopamine (DA), 5-hydroxytryptamine (5-HT), 5-hydroxyindole acetic acid (5-HIAA), 3, 4-dihydroxyphenylacetic acid (DOPAC), and homovanillic (HVA)) were investigated in CD female mice at 2, 10, 20, and 30 days post-exposure [62]. Due to the accumulation of TiO_2_ NPs in the brain, the contents of NE and 5-HT were significantly increased after exposure to 80 nm and 155 nm TiO_2_, while the concentrations of DA, DOPAC, HVA and 5-HIAA were decreased. They concluded that intranasally instilled TiO_2_ NPs could be translocated to and deposited in murine brain after absorption by nasal mucosa, and further influence the release and metabolism of monoaminergic neurotransmitters in the brain. Although these findings are intriguing, other studies are necessary to confirm these results.

Fabian *et al*. [13] investigated the tissue distribution of intravenously administered TiO_2_ NPs (70/30 anatase/rutile; 20–30 nm). Rats were treated with a single intravenous injection of a suspension of TiO_2_ NPs in serum (5 mg/kg BW), and the tissue content of TiO_2_ NPs was determined 1, 14, and 28 days later. The TiO_2_ NP levels were highest in the liver, followed in decreasing order by the levels in the spleen, lung, and kidneys, and the highest organ burdens were on day 1 post-exposure. TiO_2_ NP levels were retained in the liver for 28 days which was the duration of the experiment. There was a slight decrease in TiO_2_ NP levels from day 1 to days 14 and 28 in the spleen, and a return to control levels by day 14 in the lung and kidneys. In this study, there were no detectable levels of TiO_2_ NPs in blood cells, plasma, brain, or lymph nodes at 1, 14, and 28 days post-exposure, suggesting a rapid clearance of the TiO_2_ NPs from the blood into the lung, spleen, kidneys, and liver. TiO_2_ NPs had not been entirely cleared from the liver and spleen within the observation period, indicating that TiO_2_ NPs can accumulate in these organs after continuous intravenous exposure. It should be noted that these intravenous exposure levels were high, which may have influenced organ distribution by damaging the integrity of the endothelial barrier.

In a two week acute toxicity study [96], mice were intraperitoneally injected with different doses of TiO_2_ NPs (0, 324, 648, 972, 1296, 1944 or 2592 mg/kg BW). Examination of particle distribution demonstrated that at 1, 2, 7, and 14 days post-exposure accumulation of TiO_2_ NPs (80 nm, 100 nm, anatase) was highest in spleen, followed by liver, kidneys and lung in a decreasing order. Accumulation of TiO_2_ NPs in the spleen was the highest throughout the experimental period. Some of the particles were excreted from the kidneys (urine). These results indicated that TiO_2_ NPs could be transported to and deposited in other tissues or organs after intraperitoneal injection, although the use of extremely high intraperitoneal injection exposure doses may have affected the results. Liu *et al*. [97] investigated distribution of TiO_2_ anatase NPs (5 nm; 5, 10, 50, 100, and 150 mg/kg BW) after intraperitoneal injection in mice daily for 14 days. They found the order of the accumulation of TiO_2_ NPs in the organs was liver > kidneys > spleen > lung > brain > heart. The content of TiO_2_ NPs in the liver at the dose of 50 mg/kg was higher than that of TiO_2_ FPs at the same dose. A study by Ma *et al*. [98] found that TiO_2_ NPs (anatase, 5 nm; 5, 10, 50, 100, and 150 mg/kg BW; daily for 14 days) translocated from the site of injection, the abdominal cavity, to the brain causing oxidative stress and brain injury in ICR mice. Again, relevance of such injury is an issue due to the high exposure doses used.

These studies have shown that TiO_2_ NPs distributed to other organs after intravenous or intraperitoneal administration. Most of the NPs accumulated in the liver. TiO_2_ NPs were found in the brain after intranasal administration. However, these studies used high doses, which greatly exceed levels likely after anticipated exposures (occupational, medical, consumer use, etc.). Therefore, further investigation is necessary.

#### Metabolism

So far we have not found specific literature regarding the metabolism of TiO_2_ NPs.

#### Excretion

Similar to other inorganic NPs, TiO_2_ NPs in the systemic circulation has two potential pathways for clearance, i.e., kidneys/urine and bile/feces. The International Program on Chemical Safety for TiO_2_ shows that most ingested TiO_2_ is excreted with urine [99]. Clearance of particles from the liver *via* the bile into the feces is well known in pharmaceutics and is also postulated for TiO_2_ NPs [100]. Furthermore, every NP not absorbed by the gut epithelium will presumably be eliminated from the body *via* this pathway. Similarly, inhaled TiO_2_ NPs which are deposited in the airways of the respiratory tract and phagocytized by alveolar macrophages may be transported by mucociliary action to the larynx from where they can be cleared *via* expiration of sputum or be swallowed entering the GIT. Although NPs deposited in the alveoli can either be translocated to the interstitium, lymph nodes, or pulmonary capillaries, the majority are cleared by macrophage-mediated transport to the distal end of the mucociliary escalator. A study found that alveolar clearance for TiO_2_ FPs (5.35, 10.7, and 21.41 mg/rat; Ti IV 100% rutile; 1 μm; 7 and 42 days post-exposure) *via* macrophage phagocytosis was greater than TiO_2_ NPs (0.26, 0.52, and 1.04 mg/kg; P-25, 80/20 anatase/rutile; 21 nm) [16]. This was attributed to the higher rate of interstitialization of TiO_2_ NPs. An *in vivo* inhalation study reported similar results. They found that clearance of TiO_2_ NPs (20 nm; 7.2 × 10^6^ particles/ml; 1 h and 24 h post-exposure) from the lung by lung surface macrophages is relatively low [101]. This was based on data analysis which revealed an uptake of 0.06 to 0.12% TiO_2_ NPs by lung-surface macrophages within 24 h. Ineffective macrophage clearance of inhaled NPs from the peripheral lung would lead to bio-persistence of TiO_2_ NPs and/or favors their translocation into the lung interstitium and then to the vasculature, potentially enhancing adverse systemic effects [101]. Hydrogen-1 nuclear magnetic resonance spectroscopy (1H-NMR) was used to analyze urine metabolites of rats exposed by intratracheal instillation to low (0.8 mg/kg), medium (4 mg/kg) and high (20 mg/kg) doses of TiO_2_ NPs [102]. Significant metabolite (acetate, valine, dimethylamine, taurine, hippurate, and 2-oxoglutarate) changes were only observed in the low dose group. These compensatory changes resolve within seven days, and the results of serum biochemical assays also implied no parenchymal damages in the liver or kidney. They concluded that low dose instillation of TiO_2_ NPs resulted in a transient impact on metabolic function because the scattered NPs can migrate from the lung to liver or kidney. In contrast, TiO_2_ NPs form agglomerates at higher doses which decreases migration to systemic organs.

**Figure 2:**
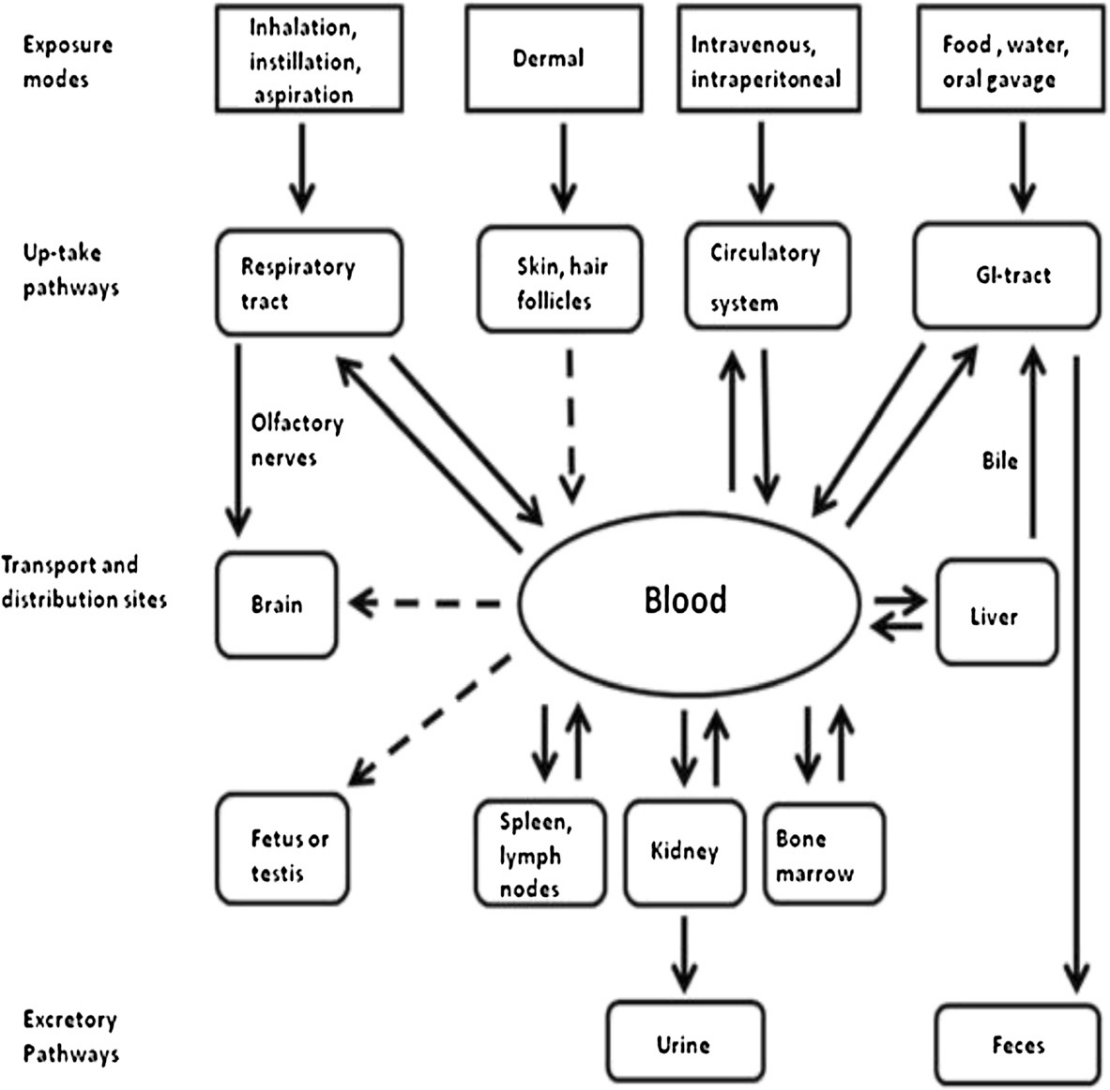
**Toxicokinetics and accumulation sites of TiO**_**2 **_**NPs.** The arrows in dotted lines represent uncertainties.

In summary, absorption, distribution, metabolism, and excretion of TiO_2_ NPs may be affected by various factors including routes of exposures and particle size, particle agglomeration and surface coating. The most frequently investigated exposure routes in the toxicokinetics studies of TiO_2_ NPs were pulmonary, lung inhalation, dermal and oral administrations. TiO_2_ NPs can be absorbed into the body through the lung and GIT. Further studies are needed to quantify the magnitude of such transport so that systemic risk can be assessed. There is no sufficient evidence available to indicate that TiO_2_ NPs have the ability to penetrate through the intact skin into the human body under normal conditions. TiO_2_ NPs injected intravenously or intra-peritoneally were found in different organs, such as liver, spleen, kidneys, lung, lymph nodes, and brain. TiO_2_ NPs may have the potential to penetrate the blood–brain barriers (BBB) and blood-placenta barriers. However, these studies employed very high doses of TiO_2_ NPs. Elimination of TiO_2_ NPs may be through kidneys/urine, and bile/feces. Though a large fraction of absorbed TiO_2_ NPs could be excreted rapidly, it is possible that not all of these particles will be eliminated from the body. As a result, accumulation of TiO_2_ NPs in some organs may take place in the human body after continuous exposure. A major site of accumulation seems to be the liver. However, there is a possibility that the accumulated TiO_2_ NPs can be completely cleared from these sites if the study time frame is increased. Therefore, further biokinetic studies are needed. Additionally, translocation of TiO_2_ NPs, at relevant lower doses, should be conducted to determine if the presence of TiO_2_ NPs at systemic sites alters their normal biological function and anatomical morphology. For example, pulmonary exposures of NPs did not cause extensive damage to the air/blood barrier, NP translocation was slow, representing less than 1% of the initial pulmonary burden at 1 week post-exposure [91]. The possible toxicokinetics of TiO_2_ NPs and accumulation sites are summarized in Figure 2.

### Toxicity of TiO_2_ NPs

The toxic effects of test substances are usually measured in terms of acute, sub-acute, sub-chronic or chronic exposure conditions. Studies with a maximum of 2 weeks (14 days) study duration are normally referred to as acute toxicity studies. Sub-acute toxicity studies last for a maximum of 4 weeks (28 days), sub-chronic toxicity studies for a maximum of 13 weeks (90 days) and chronic toxicity studies last longer than 4 months. The toxicity of TiO_2_ NPs will be discussed in terms of these types of studies.

#### Acute toxicity

Acute toxicity information for TiO_2_ NPs in humans is currently lacking. A value often given in animal toxicity studies is the median lethal dose (LD_50_)/median lethal concentration (LC_50_), which is defined as the dosage/concentration resulting in the death of 50% of the experimental animals. However, due to ethical reasons, this method of acute toxicity testing was abolished in 2002 from the Organization for Economic Co-operation and Development (OECD) acute toxicity guideline (TG 401) and is not recommended. However, there are other alternative methods such as fixed dose procedure (TG 420), up and down procedure (TG 423) and dose response method (TG 425) that can be used to determine the LD_50_. The acute toxicity studies are mentioned in the order; inhalation, intra-tracheal instillation, dermal, oral, intraperitoneal, and *in vitro*.

In an inhalation study, rats were exposed to aerosols of 0, 2, 10, or 50 mg/m^3^ TiO_2_ NPs by inhalation for 6 h/day for 5 days [103]. Necropsies were performed either immediately after the last exposure or after 3 and 16 days post-exposure. Lung inflammation was associated with dose-dependent increases in bronchoalveolar lavage fluid (BALF) total cell and neutrophil counts, total protein content, enzyme activities and levels of a number of cell mediators. No indications of systemic effects were found by measurement of appropriate clinical pathological parameters. An inhalation study on mice [104], exposed to TiO_2_ NPs (2–5 nm; 8.88 mg/m^3^; 4h/day for 10 days) showed higher counts of total cells and alveolar macrophages in the BALF. However, mice recovered by week 3 post-exposure. These inhalation studies showed that at sufficient lung burdens in both rats and mice TiO_2_ NPs can cause pulmonary inflammation. Nurkiewicz *et al*. [105] reported that inhalation of nano- or fine TiO_2_ (21 nm or 1 μm; 1.5 or 20 mg/m^3^; 24 h post-exposure) caused microvascular dysfunction in arterioles of the shoulder muscle, i.e., a failure to respond to dilators. On a mass basis, nano TiO_2_ was 6–7 times more potent than fine TiO_2._ However, the difference in potency was not present when dose was normalized to particle surface area delivered to the lungs. A recent study by the same research group found that the peripheral vascular effects associated with particulate matter (PM) exposure (TiO_2_ FP 710 nm and NP 100 nm; inhalation 1.5-16 mg/m^3^ for 4–12 h) are due to the activation of inflammatory and/or neurogenic mechanisms [106]. In addition, an increase in basal tone and a decreased responsiveness of coronary arterioles to dilators was noted 1 day after inhalation of nano TiO_2_ (21 nm; P-25:80/20 anatase/rutile; 6 mg/m^3^; at 24 h post-exposure) in another study [107]. It should be noted that microvascular dysfunction was reported at low lung burdens which did not significantly alter BALF measures of lung inflammation or damage. These findings are of interest because there are known associations between PM and cardiovascular diseases. PM exposure can result in significant changes in many cardiovascular indices, such as heart rate, heart rate variability, blood pressure, and blood coagulability [108].

Liu *et al*. [109] treated rats by intra-tracheal instillation with a single dose of 0.5, 5, or 50 mg/kg BW of TiO_2_ NPs (5, 21, or 50 nm, respectively). Rats were sacrificed one week post-exposure. Histopathological examinations of lung tissue indicated that exposure to TiO_2_ NPs induced dose-dependent inflammatory lesions in rats. In addition, on an equal mass basis, pulmonary toxicity induced by 5 nm TiO_2_ NPs was more severe than those induced by 21 and 50 nm TiO_2_ particles. The time course of pulmonary responses was reported in a study by Kobayashi *et al*. [110] at 1 and 7 days after rats were intra-tracheal instilled (19 and 28 nm; 5 mg/ml) with TiO_2_ NPs. The TiO_2_ NPs showed greater pulmonary inflammatory effect 24 h after exposure then 1 week after exposure. In these studies, the inflammatory effects of TiO_2_ NPs were locally distributed, dose dependent and recovery was probable. The effects of TiO_2_ NPs on the immune function were investigated by Liu *et al*. [111]. They evaluated immune function of rat alveolar macrophages (AM) exposed to TiO_2_ NPs (intratracheal instillation) and reported damage to the cell structure and AM dysfunction, leading to a reduction in both non-specific and specific immune responses in individuals exposed to TiO_2_ NPs (5 and 200 nm; 0.5, 5, or 50 mg/kg). The phagocytic ability of the macrophages increased when exposed to a low dose of TiO_2_ NPs and decreased when exposed to a high dose of TiO_2_ NPs. Exposure to TiO_2_ NPs also decreased the chemotactic ability of the macrophages and the expression of Fc receptors and MHC-class II on the cell surface. The mechanism responsible for these changes was mediated *via* altering nitric oxide (NO) and tumor necrosis factor-α (TNF-α) expression by the AM. The amount of NO and TNF-α secreted by macrophages was gradually increased when the dosage of TiO_2_ NPs increased. TiO_2_ NPs elicited greater NO and TNF-α production than FPs. This research group attributed the potency of TiO_2_ NPs to surface area and crystal structure [112]. The involvement of TNF-α in late airway hyper responsiveness (AHR) has recently been investigated. In a study, low pulmonary doses of TiO_2_ NPs were shown to aggravate pulmonary inflammation and AHR in a mouse model of toluene diisocyanate (TDI)-induced asthma [113]. To investigate the modulation of an asthmatic response by TiO_2_ NPs (99.9% anatase; 15 nm) in a murine model of diisocyanate-induced asthma, the investigators in this study treated mice on days 1 and 8 with 0.3% TDI or the vehicle acetone-olive oil (AOO) on the dorsum of both ears (20 μl). On day 14, the mice were oropharyngeally dosed with 40 μl of TiO_2_ NP suspension (0.8 mg/kg BW). One day later (day 15), the mice received an oropharyngeal challenge with 0.01% TDI (20 μl). On day 16, airway hyper-reactivity (AHR), BAL cell and cytokine levels, lung histology, and total serum immunoglobulin E were assessed. TiO_2_ NP exposure in sensitized mice led to a 2 fold increase in AHR, and a 3 fold increase in BAL total cell count, mainly comprising neutrophils and macrophages. Histological analysis revealed increased edema, epithelial damage and inflammation. These studies suggest TiO_2_ NPs can act as an airway irritant. Thus, if the compromised hosts are exposed to TiO_2_ NPs it may aggravate their condition.

Using acute dermal irritation studies in rabbits and the local lymph node assay in mice (CBA/JHsd mice), Warheit *et al*. [114] concluded that TiO_2_ NPs (129.4 nm in H_2_O; 80/20 anatase/rutile; 0%, 5%, 25%, 50%, or 100% TiO_2_ NPs, 3 consecutive days) were not a skin irritant or dermal sensitizer. Another study reported that acute dermal, eye and vaginal mucous membrane irritation tests revealed no significant irritation in TiO_2_ NP (size not given; 1000, 2,150, 4,640, and 10,000 mg/kg BW) treated mice at 1, 24, or 48 h post-exposure [115]. In a 14 days toxicity study, TiO_2_ NPs (20 nm; 14, 28, 42, and 56 mg/kg) applied topically on Wistar rat skin induced short term toxicity at the biochemical level [116]. Depletion in the levels of catalase and glutathione S-transferase (GST) activity were observed. In addition, there was an increase in the activity of lactate dehydrogenase (LDH) and lipid peroxidation (LPO). The levels of serum glutamic pyruvic transaminase (SGPT), serum glutamic oxaloacetic transaminase (SGOT), blood urea nitrogen (BUN), and creatinine were also increased. However, the histopathological studies did not show any observable effects at the tissue level. They concluded that short term dermal exposure to TiO_2_ NPs (42 mg/kg BW) can cause hepatic, as well as renal, toxicity in rats. The two studies cited here, in regards to dermal exposure, agree with a study mentioned earlier in the section on toxicokinetics. However, the findings of the latter study suggest that the hair follicles may be a way for TiO_2_ NPs to penetrate into live skin. It should be noted that the doses used in these studies are high and do not mimic likely human exposures.

Warheit *et al*. [114] reported in acute oral toxicity studies that TiO_2_ NPs (129.4 nm in H_2_O; 175, 550, 1750 or 5000 mg/kg; 80/20 anatase/rutile; 48 h intervals for 14 days) were very low in toxicity and produced short-term and reversible ocular conjunctiva redness in rabbits. In another study, the acute toxicity in mice was investigated after a single oral administration of TiO_2_ particles (25, 80, and 155 nm; 5 g/kg BW) [59]. Over two weeks post-exposure, TiO_2_ particles showed no obvious acute toxicity. However, the female mice showed high hepatic coefficients in the 25 and 80 nm groups. The changes of serum biochemical parameters (alanine aminotransferase (ALT), aspartate aminotransferase (AST), LDH, and BUN), and pathology of the liver and kidney indicated that hepatic renal injury was induced after exposure. Even though there were significant changes of serum LDH in TiO_2_ NP (25 and 80 nm) treated animals, indicating the presence of myocardial damage, the pathology results for the heart, lung, testicles (ovary), or spleen showed no abnormal changes. These oral exposure studies showed biochemical changes, but systemic toxicity was not demonstrated.

Intraperitoneal studies may be done to address the effects of possible TiO_2_ NP use in nanomedicine. At the higher doses of an intraperitoneal exposure study done on mice, TiO_2_ NPs (anatase, 5 nm; 5, 10, 50, 100, and 150 mg/kg BW; everyday for 14 days) caused serious damage to the liver, kidneys, and myocardium and disturbed the balance of blood sugar and lipid [97]. Furthermore, with increasing doses of TiO_2_ NPs, indicators of liver function, such as ALT, leucine acid peptide, pseudocholinesterase, total protein, and albumin levels, were enhanced significantly; the indicators of kidney function, such as uric acid and BUN, were decreased; and the activities of AST, creatine kinase (CK), LDH, and alpha-hydroxybutyrate dehydrogenase, indicators of the myocardium function, were increased. The contents of triglycerides, glucose, and high-density lipoprotein cholesterol were significantly elevated. The authors concluded that the accumulation of TiO_2_ NPs in the organs might be closely related to the differences in the coefficients of organs and the inflammatory responses of mice. In addition, they reported that the LD_50_ value of TiO_2_ NPs through intraperitoneal injection in mice was 150 mg/kg BW. Mice intraperitoneally injected with TiO_2_ NP (50 nm; 0, 324, 648, 972, 1296, 1944 or 2592 mg/kg; 24 h, 48 h, 7 days, and 14 days) showed signs of acute toxicity, such as passive behavior, loss of appetite, tremor, and lethargy. Slightly elevated levels of ALT and AST were observed. Histopathological examinations showed that some TiO_2_ NPs entered the spleen and caused lesions. Thrombosis was found in the pulmonary vascular system. In addition, hepatocellular necrosis and apoptosis, hepatic fibrosis, renal glomerular swelling and interstitial pneumonia associated with alveolar septal thickening were also observed in the high dose group. Ma *et al*. [117] stated that inflammatory responses and liver injury may be involved in TiO_2_ NP (5 nm; 5, 10, 50, 100, and 150 mg/kg BW; everyday for 14 days) induced liver toxicity. The real-time quantitative PCR (RT-PCR) and enzyme-linked immunosorbent assay (ELISA) analyses showed that TiO_2_ NPs can significantly alter the mRNA and protein expression of several inflammatory pathways, including nuclear factor kappa-light-chain-enhancer of activated B cells (NF-κB), macrophage migration inhibitory factor (MMIF), TNF-α, interleukin(IL)-6 (IL-6), IL-1β, cross-reaction protein, IL-4, and IL-10. In addition to this, we have already mentioned some studies [19,25,118] that have also reported the inflammatory effects of TiO_2_ NPs. TiO_2_ NPs also induce some neurons to turn into filamentous shapes and others into inflammatory cells after translocating from the abdominal cavity [98]. Oxidative stress and injury of the brain triggered a cascade of reactions, such as LPO, decreases of the total anti-oxidation capacity and activities of antioxidative enzymes, the excessive release of NO, the reduction of glutamic acid, and the down-regulated level of acetylcholinesterase activity. The acute toxicity of intraperitoneally injected TiO_2_ NPs is systemic, it involves pathological and biochemical effects on the liver, kidney, heart and brain.

Since *in vitro* studies can be used as predictive indicators of acute toxicity, some are mentioned here. The influence TiO_2_ NPs on erythrocytes was systematically investigated by Li *et al*. [119]. Their results indicate that erythrocytes treated with TiO_2_ NPs underwent abnormal sedimentation, hemagglutination and dose dependent hemolysis, which totally differed from cells treated with TiO_2_ FPs. Another study reported that hemolysis (washed human erythrocytes, 37°C incubation for 1 h) caused by TiO_2_ FPs was 73 times greater than TiO_2_ NPs [120]. However, the hemolysis was abolished by plasma, indicating that in *in vivo* conditions the presence of plasma may prevent hemolysis. A study on mouse macrophages (Ana-1 and MH-S cells) found that TiO_2_ NPs (5, 10, 25, and 100 nm; anatase) caused low toxicity to MH-S cells [121]. Another study found TiO_2_ NPs (25 and 80 nm; 0, 10, 20, 40, and 80 mg/l; 24 h) inhibited gap junction intracellular communication between lung fibroblasts [122]. A number of *in vitro* studies also show toxic effects of TiO_2_ NPs on cells of the circulatory system.

In summary, the acute toxicity of TiO_2_ NPs have been frequently studied in rat and mouse models following multiple exposure routes of administration. The number of studies targeting the respiratory system outweighs the other exposure routes. Studies exposing the pulmonary system to TiO_2_ NPs produced both local and systemic symptoms and aggravate pre-existing symptoms. TiO_2_ NPs administered through the lung are more inflammatory than FPs of similar chemistry at equal mass concentrations. However, on an equal particle surface area basis, pulmonary inflammation to TiO_2_ NPs was similar to that of TiO_2_ FPs. The results from the other exposure routes cannot be ignored. For example, research evidence demonstrates that TiO_2_ NPs can be absorbed through the lung or GIT into the systemic circulation and then distributed in different organs such as the liver, kidneys, spleen, or even the brain. Distribution and accumulation of TiO_2_ NPs in the organs could induce organ injuries and inflammatory responses. However, most of the doses employed are too high to be realistic in occupational settings. *In vitro* studies also show effects of TiO_2_ NPs on the blood circulation system.

#### Sub- acute toxicity

Silicon dioxide (SiO_2_)-coated rutile TiO_2_ NPs (40 nm; 10 mg/m^3^; 2 h on 4 consecutive days, or 2 h on 4 consecutive days, 4 weeks) caused pulmonary neutrophilia, increased expression of TNF-α and neutrophil attracting chemokine (CXCL 1) in lung tissues [123]. However, they attributed the effects to the surface coating with SiO_2_. Others observed that TiO_2_ NPs caused minimal inflammatory changes in the lungs, leucopenia, and a decrease in β-glucuronidase after inhalation [92].

In another study [124], rats were intra-tracheally instilled with TiO_2_ NPs (1 or 10 mg/kg BW). At 10 mg/kg BW, LDH activity (1, 7, 14, and 28 days), malodialdehyde (MDA) (1, 7, and 14 days), total protein (1 and 7 days), as well as, the number of leukocytes (1 and 7 days) were all increased significantly when compared with the controls. Histopathological examination revealed a marked increase of pulmonary inflammation in the lungs in 10 mg/kg BW treated rats. While investigating dose–response relationships for intra-tracheal instillation of TiO_2_ NPs (20 nm) and FPs (250 nm), Oberdorster *et al*. [125] observed a significant pulmonary inflammatory response to TiO_2_ NPs in rats and mice, which included an increase of total protein in BALF, LDH activity, and acid-glucosidase. They concluded that the greater toxicity of the TiO_2_ NPs correlated well with their greater surface area per mass. Li *et al*. [79] investigated the effects of TiO_2_ NPs (3 nm) in mice after intra-tracheal instillation at a total dose of 13.2 mg/kg BW (once a week for 4 weeks). At 28 days after instillation, they found that instilled TiO_2_ NPs could induce lung damage, and change the permeability of the alveolar-capillary barrier. TiO_2_ NPs were able to access the blood circulation and reach extra-pulmonary tissues, such as the liver and kidneys, leading to different levels of tissue injury. In addition, TiO_2_ NPs might pass through the BBB and induce the injury through an oxidative stress response. At other sites, TiO_2_ NPs (1.0, 0.5, and 0.1 mg/ml; twice/week for 6 weeks) caused dyslipidemia and accelerated the development of atherosclerosis and plaque rupture in intra-tracheally instilled ApoE−/−mice [126]. In this study, viscera index, blood total cholesterol (TC), triglyceride (TG), high density lipoprotein cholesterol (HDL-C), low density lipoprotein cholesterin (LDL-C), and organic lipid ratio were assessed as biomarkers. Artery and aortic root issues were assessed by histopathology. Another study investigated whether photocatalytic TiO_2_ NPs (28 nm; rutile; 2, 10, 50, or 250 μg) exhibited an adjuvant effect, when administered through intraperitoneal injection in combination with ovalbumin (OVA) in mice [127]. The mice in this study were treated with OVA, OVA + TiO_2_ NPs or OVA + AlOH_3_ and challenged with aerosols of OVA. The TiO_2_ NPs promoted a Th2 dominant immune response with high levels of OVA-specific IgE and IgG1 in the serum, and influx of eosinophils, neutrophils and lymphocytes in BALF. Significantly higher levels of OVA-specific IgE were induced by TiO_2_ NPs than the standard adjuvant, AlOH_3_. However, the two substances were comparable regarding the level of eosinophilic inflammation and interleukins present in BALF.

The oral toxicological effects of TiO_2_ NPs (dosed at 0.16, 0.4, and 1 g/kg) were investigated using conventional approaches and metabolomic analysis in Wistar rats [128]. The urine and serum were analyzed by 1H-NMR using principal components and partial least squares discriminant analyses. The metabolic signature of urinalysis in TiO_2_ NPs-treated rats showed increases in the levels of taurine, citrate, hippurate, histidine, trimethylamine-N-oxide (TMAO), citrulline, alpha-ketoglutarate, phenylacetylglycine (PAG) and acetate. Decreases in the levels of lactate, betaine, methionine, threonine, pyruvate, 3-D-hydroxybutyrate (3-D-HB), choline and leucine were also observed. The metabolomics analysis of serum showed increases in TMAO, choline, creatine, phosphocholine and 3-D-HB as well as decreases in glutamine, pyruvate, glutamate, acetoacetate, glutathione and methionine after TiO_2_ NPs treatment. AST, CK and LDH were elevated and mitochondrial swelling in heart tissue was observed in TiO_2_ NPs-treated rats. They concluded that their findings indicated that disturbances in energy and amino acid metabolism, and the gut microflora environment may be attributable to slight injury to the liver and heart caused by TiO_2_ NPs. They proposed that the NMR-based metabolomic approach may be a reliable and sensitive method to study the biochemical effects of nanomaterials. What one can crudely deduce from these findings is that, in terms of occupational exposures, those with underlying health issues such as asthma and heart disease may be at risk of TiO_2_ NPs toxicity. However, since the studies were conducted in animals, there is a need for epidemiological studies in the workplace, to quantify the risks of TiO_2_ NPs in the workplace. This is further discussed in part **IX**, the *epidemiological studies* section.

#### Sub-chronic toxicity

A subchronic inhalation study comparing pulmonary responses to TiO_2_ NPs in several species was performed [129]. Female rats, mice, and hamsters were exposed to aerosol concentrations of 0.5, 2.0, or 10 mg/m^3^ TiO_2_ NPs (P-25; 21 nm; 6 h/day, 5 days/week, for 13 weeks). At each time point, TiO_2_ NPs burdens in the lung and lymph nodes and selected lung responses were examined. Retained lung burdens increased in a dose-dependent manner in all three species and were at a maximum at the end of exposures. There were significant species differences in the pulmonary responses to inhaled TiO_2_ NPs. Under conditions where the lung TiO_2_ NPs burdens were equivalent, rats developed a more severe inflammatory response than mice and, subsequently, developed progressive epithelial and fibroproliferative changes. Clearance of particles from the lung was markedly impaired in mice and rats exposed to 10 mg/m^3^ TiO_2_ NPs, whereas clearance in hamsters did not appear to be affected at any of the administered doses.

Warheit *et al*. [130] intra-tracheally instilled TiO_2_ NPs (25 and 100 nm; 1 or 5 mg/kg BW; 24 h, 1 week and 3 months) into rats to compare several types of TiO_2_ FPs and NPs with different sizes, surface areas, and crystal structures. In the comparison among these particles, even though the difference in surface areas was as large as 30 fold, the observed lung inflammatory responses were almost the same for the two particle sizes. They, therefore, concluded that toxicities of TiO_2_ particles through lung instillation are not dependent upon particle size and surface area. In addition, the same research group suggested that the toxicity was dependent on particle surface properties instead of surface areas. Roursgaard *et al*. [131] intratracheally instilled mice with single fixed doses of 5, 50, and 500 μg of TiO_2_ FPs and NPs (rutile). They found, in the acute phase, both FPs and NPs induced elevation of IL-6 and total protein in BALF at the highest doses. Similar effects were observed in acute (24 h) and sub-chronic (3 months) airway inflammation for two different sizes of TiO_2_ particles. These results suggest that TiO_2_ NPs may not be more cytotoxic or cause more inflammation to the lungs compared to FPs of similar composition. However, the results in both the Warheit *et al*. and Roursgaard *et al*. studies may be questioned due to poor dispersion of TiO_2_ NPs as suggested by Sager *et al*. [16]. Indeed, structural sizes of the different particles as delivered to the rats did not sifnificantly differ.

Wang *et al*. [132] investigated the influence of intranasally instilled TiO_2_ NPs on monoaminergic neurotransmitters at different times post-exposure (25, 80, and 155 nm; 50 mg/kg; 2, 10, 20, and 30 days) in CD female mice. They used ICP-MS to analyze the TiO_2_ NP contents in murine brain. The monoaminergic neurotransmitters such as NE, DA, 5-HT, 5-HIAA, DOPAC, and HVA, were determined by reversed-phase high performance liquid chromatography (RP-HPLC) with an electrochemical detector. TiO_2_ NPs in murine brain increased after 10 days for the 25 nm group ((1059.3+/−293.5) ng/g). It declined slowly at 20 days post-exposure ((654.7+/−269.2) ng/g). At 30 days post-exposure, the TiO_2_ NPs content remained the same as at 20 days. Due to the accumulation of TiO_2_ NPs in the brain, the levels of NE and 5-HT increased significantly after exposure to 80 and 155 nm TiO_2_ NPs, while decreases in the levels of DA, DOPAC, HVA and 5-HIAA were observed. The inhaled TiO_2_ NPs could be translocated to and deposited in murine brain after absorption through the nasal mucosa, and could influence the release and metabolism of monoaminergic neurotransmitters in brain.

Wu *et al*. [133] investigated the penetration and potential toxicity of TiO_2_ NPs after *in vitro* (porcine ears) and *in vivo* animal (domestic pig ears, BALB/c hairless mice) dermal exposure. They concluded that TiO_2_ NPs (various sizes) cannot penetrate through the SC 24 h after exposure to isolated porcine skin. However, after being topically applied on pig ear *in vivo* for 30 days, TiO_2_ NPs (4 and 60 nm; 24 mg of 5% TiO_2_ on an area of 3 cm^2^) could penetrate through the horny layer, and be located in the deep layer of the epidermis. Moreover, after 60 days dermal (400 μg/cm^2^) exposure in hairless mice, TiO_2_ NPs not only penetrated the skin, but also reached different tissues and induced diverse pathological lesions in several major organs. In addition, they found TiO_2_ NPs (21 nm, P-25) in the mouse brain without inducing any pathological changes.

Hu *et* al. [134] intragastrically instilled ICR mice with TiO_2_ NPs (5 nm anatase; 0, 5, 10, and 50 mg/kg BW; every day for 60 days). Their aim was to determine whether TiO_2_ NPs exposure results in persistent alterations in nervous system function. The Y-maze test showed that TiO_2_ NPs exposure could significantly impair the spatial recognition memory. TiO_2_ NPs also caused disturbances of the homeostasis of trace elements, enzymes and neurotransmitter systems in the mouse brain. They also found that there were significant alterations in the contents of Ca, Mg, Na, K, Fe, and Zn in the brain. TiO_2_ NPs also significantly inhibited the activities of Na^+^/K^+^-ATPase, Ca_2_^+^-ATPase, Ca_2_^+^/Mg_2_^+^-ATPase, acetylcholine esterase, and nitric oxide synthase (NOS). The contents of some monoamines neurotransmitters, such as NE, DOPAC, 5-HT and its metabolite 5-HIAA, were significantly decreased, while acetylcholine, glutamate, and NO were significantly increased.

#### Chronic toxicity (excluding carcinogenicity)

In work environments, the potential chronic toxicity of TiO_2_ NPs is likely to be of more concern than acute effects. Early studies suggest that TiO_2_ is not highly toxic.

Chronic lung inhalation studies [9,135] that exposed pigs or rats, respectively, to TiO_2_ FPs have reported findings of pulmonary pathology such as increased incidences of pneumonia, squamous metaplasia [135], sustained pulmonary responses [136], enhanced proliferation of pulmonary cells, defects in macrophage function [137], alveolar epithelial metaplasia, progressive fibroproliferative lesions [138] and accumulation of macrophages in interalveolar septa [9]. Some studies on TiO_2_ NPs show similar effects. Oberdorster *et al*. [15] investigated the correlation between particle size, *in vivo* particle persistence, and lung injury after a 12 week inhalation (23.5±2.9 mg/m^3^) experiment in rats (Fischer 344) exposed to TiO_2_ particles (20 and 250 nm). They reported inflammation and lung injury and concluded that the greater pulmonary effects of NPs, compared to FPs, can be explained by their larger specific surface area, the greater interstitial access, and their altered biopersistence, resulting in increased retention of NPs.

Exposure to TiO_2_ NPs (5–6 nm) resulted in chronic spleen injury, in a 90 day study done on ICR mice (intragastric administration; 2.5, 5, and 10 mg/kg; everyday) [139]. Blood cells, platelets, hemoglobin, immunoglobulin and lymphocyte subsets (such as CD3, CD4, CD8, B cell, and natural killer cell) of mice were significantly decreased. There was also a significant increase in the levels of NF-κB, TNF-α, MMIF, IL-2, IL-4, IL-6, IL-8, IL-10, IL-18, IL-1β, cross-reaction protein, transforming growth factor-β (TGF- β), interferon-γ, Bax, and CYP1A1 expression, and decreases in the levels of Bcl-2 and heat shock protein 70 (Hsp70) expression. Long-term exposure to low dose TiO_2_ NPs may cause spleen injury, resulting from alteration of inflammatory and apoptotic cytokines expression and reduction of immune capacity.

In conclusion, TiO_2_ NPs exhibit moderate toxicity, inducing pulmonary inflammatory response and enhanced proliferation of pulmonary cells at relatively high doses. TiO_2_ NPs are found to induce greater pulmonary inflammatory effects compared to TiO_2_ FPs. The modulatory effects of TiO_2_ NPs in asthmatic responses need to be confirmed. As evident in the acute toxicity studies, the chronic toxicity studies also focus on the respiratory system. However, with the increase in consumer use of sunscreens that contain TiO_2_ NPs, more effort should be put into carrying out chronic exposure studies for topically applied consumer goods.

In all the different types of toxicity study conditions, pulmonary toxicity seems to be a common finding. The number of studies on pulmonary toxicity also outweighs studies of other exposure routes, emphasizing its importance especially in reference to environmental and occupational exposures. Most of these studies also show that the endpoints of oxidative stress and inflammation seem to be most affected. This mechanistic information can be helpful in increasing the specificity and sensitivity of future *in vitro* and *in vivo* studies.

### Genotoxicity

The genotoxicity of TiO_2_ NPs remains controversial [140]. Early studies suggest that TiO_2_ is not genotoxic in standard assays [141]. In recent years, *in vivo* and *in vitro* studies have examined the genotoxicity of TiO_2_ NPs. Test systems used frequently in *in vivo* studies of genotoxicity of TiO_2_ NPs include rat or mouse bone marrow cells. End points used in the *in vitro* studies include micronucleus (MN) test, Ames test, mammalian cell gene mutation, DNA breaks, chromosomal alterations, and cell transformation. These genotoxicity endpoints provide useful data for hazard identification of TiO_2_ NPs.

#### In vivo studies

A few *in vivo* studies have been carried out to investigate the genotoxicity of TiO_2_ NPs. A study by Yazdi *et al*. [142] found that inhalation of TiO_2_ NPs provoked lung inflammation which was strongly suppressed in IL-1R– and IL-1α– deficient mice. They concluded that the inflammation caused by TiO_2_ NPs *in vivo* was driven by IL-1α. The signaling of IL-1R by TiO_2_ NPs is similar to that of asbestos. TiO_2_ NPs (6 mg/m^3^; 4 h) also increased the phosphorylation of p38 and troponin 1 in cardiac muscle of rats exposed through inhalation, at 1 day post exposure [143]. These inhalation studies show that TiO_2_ NPs can affect the expression of certain genes in both the heart and lung.

An intratracheal instillation study showed that hypoxanthine phosphoribosyltransferase (HPRT) mutation frequency was increased in alveolar type II cells from rats exposed to TiO_2_ NPs (anatase; 18 nm; 100 mg/kg; 15 months) [144]. While in a study by Trouiller *et al*. [32], TiO_2_ NPs were genotoxic, clastogenic and caused moderate inflammation *in vivo* in mice exposed through drinking water (21 nm, P-25). TiO_2_ NPs at 500 mg/kg BW induced both DNA single and double-strand breaks and chromosomal damage. TiO_2_ NPs induced 8-hydroxy-2^′^-deoxyguanosine, γ-H2AX foci, micronuclei, and DNA deletions. The formation of γ-H2AX foci is indicative of DNA double-strand breaks. They suggested that TiO_2_ NPs–induced genotoxicity *in vivo* in mice is possibly caused by a secondary genotoxic mechanism associated with inflammation and/or oxidative stress. RT-PCR and ELISA analysis showed that intra-gastrically administered TiO_2_ NPs (anatase; 5 nm; 5, 10, and 50 mg/kg; everyday for 60 days) significantly increased mRNA and protein expression of Toll-like receptor-2 (TLR2), TLR-4, IκB kinases (IKK-α, IKK-β), NF-κB, NF-κBP52, NF-κBP65, TNF-α, and NF-κB-inducible kinase (NIK), and decreased the expression of IκB and IL-2 in mice [145]. They stated that the signaling pathway of liver injury in the TiO_2_ NPs-stimulated mouse liver sequentially might occur *via* activation of TLRs → NIK → IκB kinase → NF-κB → TNF-α → inflammation → apoptosis → liver injury. Another intra-gastric administration study focusing on the molecular mechanisms of kidney injury of mice, found that TiO_2_ NPs (5–6 nm; 2.5, 5, and 10 mg/kg; everyday for 90 days) activated NF-κB, leading to increased expression of TNF-α, MMIF, Il-2, Il-4, Il-6, Il-10, Il-18, Il-1β, cross-reaction protein, TGF- β, interferon-γ, and CYP1A1, and a decrease in Hsp70 [146]. This showed that TiO_2_ NPs accumulate in the kidney, causing nephric inflammation, cell necrosis and dysfunction. Activation of NF-κB and increases in the expression of similar inflammatory cytokines were observed in a study by Sun *et al*. [147] after intratracheal instillation of TiO_2_ NPs (2.5, 5, and 10 mg/kg; 90 days) in mice. In addition, an increase in hemeoxygenase-1 (HO-1) expression and a decrease in NF-κB-inhibiting factor and Hsp70 expression were also observed. They suggested that the generation of pulmonary inflammation caused by TiO_2_ NPs (5–6 nm) in mice is closely related to oxidative stress and the expression of inflammatory cytokines. TiO_2_ NPs effectively activated caspase-3 and -9, decreased gene and protein levels of Bcl-2, Bax and cytochrome c, and promoted ROS accumulation in mice spleen [148]. In this study mice were intraperitoneally injected with TiO_2_ NPs for 45 days consecutively. TiO_2_ NPs accumulated in the mouse spleen, leading to congestion and lymph nodule proliferation of spleen tissue, and splenocyte apoptosis. Taken together, this study indicated that TiO_2_ NPs induce apoptosis in the mouse splenocyte *via* mitochondrial-mediated pathway.

In regards to *in utero* genotoxicity, Jackson *et al*. [149] analyzed hepatic gene expression in newborns of C57BL/6BomTac dames exposed to TiO_2_ (surface coated UV-Titan; 1 h/day; 42 mg UV-Titan/m^3^) using DNA microarrays. UV-Titan exposure did not induce DNA strand breaks in time-mated mice or their offspring. Even though there were changes in the expression of genes related to retinoic acid signaling in the females as indicated by transcriptional profiling of newborn livers. They concluded that the changes may be a secondary response to maternal inflammation although no direct link was evident through gene expression analysis. Another inhalation study on female C57BL/6BomTac mice (UV-Titan; 20 nm; 1h/day for 11 consecutive days; 42.4±2.9 mg surface-coated nanoTiO_2_/m^3^; sacrificed 5 days following the last exposure) showed that nanoTiO_2_ exposure resulted in increased levels of mRNA for acute phase markers. serum amyloid A-1 (Saa1) and serum amyloid A-3 (Saa3), several CXC and CC chemokines, and cytokine tumor necrosis factor genes [150]. Further protein analysis of Saa1 and 3 showed selective up-regulation of Saa3 in lung tissues. They also showed the up-regulation of miR-1, miR-449a and a 60-fold induction of miR-135b. They concluded that inhalation of surface-coated nanoTiO_2_ results in changes in the expression of genes associated with acute phase, inflammatory and immune response 5 days post exposure with concomitant changes in several miRNAs.

However, not all studies showed genotoxic effects. A study investigating the effects of NPs on the female germline found that TiO_2_ NPs (UV-Titan) do not induce expanded simple tandem repeat (ESTR) *loci* mutations in the germline of prenatally exposed female mice [151]. In this study, pregnant C57BL/6 mice were exposed by whole-body inhalation to the TiO_2_ NPs (UV-Titan L181; ~42.4mg/m^3^) on gestation days (GD) 8–18. F2 descendents were collected and ESTR germline mutation rates in this generation were estimated from full pedigrees (mother, father, and offspring) of F1 female mice (192 UV-Titan-exposed F2 offspring and 164F2 controls). Most of these studies were carried out on C57BL/6 mice strains. The positive endpoints of interest were polychromatic erythrocyte (PCE) micronuclei, γ-H2AX foci formation, DNA damage, HPRT mutation frequency and mRNA expression. However, some PCE micronuclei and ESTR mutations were not positive. An inhalation exposure (C57BL/6J mice; treated with 0.8, 7.2, and 28.5 mg/cm^3^ for 5 days; 4h/day) study by Lindberg *et al*. [152] showed no significant effect on the level of DNA damage in lung epithelial cells or micronuclei in bone marrow polychromatic erythrocytes (PCE’s) by freshly prepared TiO_2_ NPs (74% anatase; 26% brookite). In addition, the rate of PCE MN cells induced in mice after oral administration of TiO_2_ NPs (1, 2, and, 5 g/kg BW) were also not significantly different from controls [115]. Another study stated that DNA adduct formation in rat lungs was not detected following chronic inhalation for two years to TiO_2_ NPs (10.4 mg/m^3^) [153].

The *in vivo* genotoxicity studies targeted different organs as well as the reproductive system show that the TiO_2_ NPs increased the expression of the inflammatory cytokines, the mRNA expression of toll like receptors, gene mutations of the HPRT, induction of γ-H2AX foci, DNA deletions, and PCE. Increases in expression of HO-1, NF-κB, and Hsp70 were also observed. However, some studies also show that TiO_2_ NPs were not genotoxic. These disparities in results may be due to the differences in the physicochemical characteristics of the TiO_2_ NPs used, or the exposure metrics used by the investigators.

#### In vitro studies

Many *in vitro* studies have been conducted to investigate the genotoxicity of TiO_2_ NPs. TiO_2_ NPs have also been observed around the nuclei in the vicinity of the endoplasmic reticulum in cultured human-derived retinal pigment epithelial cells (ARPE-19) after exposure to high concentrations (30 μg/ml) [154]. The studies that have been conducted have tried to compare effects TiO_2_ NPs according to particle sizes, surface coatings, crystal structure, dose ranges, different cell lines, and exploratory studies.

Recently, a study by Jugan *et al*. [155] has shown that spherical TiO_2_ NPs (12–140 nm; both anatase and rutile) can induce single strand breaks, oxidative lesions to DNA and oxidative stress in A549 cells. They also showed that TiO_2_ NPs impair the cell’s ability to repair DNA by deactivation of both nucleotide excision repair (NER) and base excision repair (BER) pathways. Others have also found that TiO_2_ NPs cause increased extracellular ROS, HO-1, and NOS mRNA expression and TNF-α release in NR8383 rat lung alveolar macrophages [93]. TiO_2_ NPs demonstrated cytotoxic and genotoxic effects in human amnion epithelial (WISH) cells in another recent study [156]. In this study, polyhedral rutile TiO_2_ NPs (30.6 nm; 20 μg/ml) caused a 14 fold increase in olive tail moment (OTM), while cells treated with 0.625-10 μg/ml exhibited significant reduction in catalase activity and GSH level. There was a 1.87 fold increase in intracellular ROS generation and 7.3% increase in G_2_M cell cycle arrest.

Bhattacharya *et al*. [157] reported that human lung fibroblasts were more sensitive regarding cyto- and genotoxic effects caused by TiO_2_ NPs than human bronchial epithelial (BEAS-2B) cells. In this study, TiO_2_ NPs induced oxidative stress and DNA-adduct formation (8-OHdG) but not DNA-breakage in human lung fibroblasts. Hamster lung fibroblasts (V79 cells) were used in a study focusing on cyto- and genotoxic effects of TiO_2_ NPs (untreated anatase; 30–50 nm) and vanadium pentoxide (V_2_O_5_)-treated anatase particles [158]. V_2_O_5_-treated TiO_2_ NPs were capable of inducing greater DNA damage in mammalian cells through production of free radicals than untreated particles. V_2_O_5_-treated TiO_2_ NPs formed pronounced acellular and cellular radicals of interest. Surface-treated TiO_2_ NPs particles coated with V_2_O_5_ are used industrially for selective catalytic reactions such as the removal of nitrous oxide from exhaust gases of combustion power plants (SCR process) and in biomaterials for increasing the strength of implants.

Wang *et al*. [30] detected genotoxicity of TiO_2_ NPs in cultured human lymphoblastoid cells using the cytokinesis block micronucleus (CBMN) assay, the Comet assay, and the HPRT gene mutation assay. The cells were incubated for 6, 24 and 48 h with 0, 26, 65 or 130 μg/ml TiO_2_ NPs (7–8 nm). TiO_2_ NPs induced approximately a 2.5-fold increase in the frequency of micronucleated/binucleated cells (130 μg/ml), approximately a 5-fold increase in tail moment (65 μg/ml), and approximately a 2.5 fold increase in the HPRT mutation frequency (130 μg/ml). TiO_2_ anatase NPs and larger rutile particles provoked higher IL-1β production in macrophage-like human THP-1 cells [159]. A study on human monoblastoid cell line (U937) found that TiO_2_ NPs (<100 nm) induced both apoptotic and necrotic modifications at exposures of 0.005-4 mg/ml for 24 and 48 h [160]. Another study aimed at validating *in vitro* test systems for apoptosis induced by NPs found that TiO2 NPs induced DNA fragmentation in RAW264.7 macrophages [161]. While in cultured human lymphocytes TiO_2_ NPs increased the proportion of sub-G1 cells, activated caspase-9 and caspase-3, and induced caspase-3-mediated PARP cleavage [162]. Time-sequence analysis of the induction of apoptosis revealed that TiO_2_ NPs triggered apoptosis through caspase-8/Bid activation. They stated that TiO_2_ NPs induced apoptosis is mediated by the p38/JNK pathway and the caspase-8-dependent Bid pathway in human lymphocytes. However, in a study conducted on BEAS 2B cells, Shi *et al*. [163] noted that TiO_2_ NPs induced apoptosis *via* the mitochondrial apoptosis pathway independent of caspase 8/t-Bid pathway. These results show that different cell lines exhibit different responses to TiO_2_ NPs.

Another study on human lymphocytes showed that, TiO_2_ NPs significantly increased MN formation and DNA breakage [164]. The generation of ROS in TiO_2_ NP-treated cells was also observed. N-acetylcysteine (NAC) supplementation inhibited the level of TiO_2_ NP-induced DNA damage. The inhibitive nature of NAC on ROS formation in cells exposed to TiO_2_ was also noted by Xue *et al*. [165]. Shukla *et al*. [28] also demonstrated ROS involvement in oxidative DNA damage and MN formation in human epidermal cells. Ghosh *et al*. [166] investigated the genotoxicity of TiO_2_ NPs in plant and human lymphocytes using classical genotoxic endpoints: Comet assay and the DNA laddering technique. TiO_2_ NPs were found to be genotoxic at a low dose of 0.25 mM followed by a decrease in the extent of DNA damage at higher concentrations. In contrast, TiO_2_ FPs were consistently genotoxic at doses of 1.25 mM and above. This study concluded that the TiO_2_ NPs possess genotoxic potential in plant and human lymphocytes. These results imply that genotoxic effects of TiO_2_ NPs may occur through ROS generation in lymphocytes.

Uncoated TiO_2_ anatase NPs (99.7%; <25 nm) and TiO_2_ rutile FPs (99.9%; <5 μm) were shown to be more efficient than SiO_2_-coated TiO_2_ rutile NPs (>95%, <5% amorphous SiO_2_ coating; 10×40 nm) in inducing DNA damage, whereas only TiO_2_ anatase NPs were able to slightly induce micronuclei in a study by Falck *et al*. [167] on BEAS 2B cells (1–100 μg/cm^2^; 24, 48, and 72 h). The lower activity of nano sized rutile in genotoxicity is likely due to its coating. This conclusion is supported by Mano *et al*. [168]. They found that when TiO_2_ NPs (P25; 25 nm; 80/20 anatase/rutile) were coated with polyethylene glycol (PEG), their cytotoxic effects and induction of stress related genes in human pulmonary epithelial (NCI-H292) cells and human acute monocytic leukemia (THP-1) cells significantly decreased. Analysis of mRNA expression indicated that the expression of particular biomarkers depends upon the cell type, and that modification of TiO_2_ NPs with PEG reduces their cytotoxicity and reduces the induction of genes associated with stress and toxicity.

Petkovic *et al*. [21] investigated the genotoxic responses to two types of TiO_2_ NPs (<25 nm anatase: TiO_2_-An) and (<100 nm rutile: TiO_2_-Ru) in human hepatoma HepG2 cells. They found that TiO_2_-An, caused a persistent increase in DNA strand breaks (Comet assay) and oxidized purines (Fpg-Comet). Both types transiently upregulated mRNA expression of p53 and its downstream regulated DNA damage responsive genes (mdm2, gadd45α, and p21). A recent study conducted with Caco-2 cells found that in contrast to pure anatase TiO_2_ NPs, anatase/rutile TiO_2_ NPs induced significant LDH leakage and mild DNA damage as shown by the fpg-Comet assay [169]. The anatase/rutile NPs also showed higher toxicity per unit surface area. The investigators used the WST-1 assay to show that there was highly significant correlation between the specific surface area of anatase and cytotoxicity.

Gurr *et al*. [170] investigated the oxidative damage induced by TiO_2_ NPs in the absence of photo-activation in BEAS 2B cells. Results indicated that TiO_2_ NPs (anatase; 10 and 20 nm) in the absence of photo-activation induced oxidative DNA damage, LPO, and micronuclei formation. However, treatment with TiO_2_ FPs (anatase; >200 nm) did not. Huang *et al*. [171] investigated the cell transformation mediated by long-term exposure to TiO_2_ NPs and found that TiO_2_ NPs not only increased cell survival and growth in soft agar but also the numbers of multinucleated cells and MN. To study the potential of fine (>200 nm) and nano TiO_2_ particle (≤20 nm) to induce chromosomal changes, Rahman *et al*. [172] treated SHE cells with 1.0 μg/cm^2^ of TiO_2_ particle for 12–72 h. The micronuclei assay revealed a significant increase in MN induction in SHE cells after NP treatment, whereas TiO_2_ FPs did not show significant induction of MN formation. However, other investigators who used the same cell line have stated that cytotoxicity and genotoxicity induced by metal oxide NPs are not always higher than those induced by their FP counterparts (14–35 nm; 5,10, and 50 μg/cm^2^) [173]. Lu *et al*. [140] found TiO_2_ particles (sizes not reported) to be toxic to Chinese hamster ovary-K1 (CHO-K1) cells. The sister chromatid exchange (SCE) frequency and MN frequency in CHO-K1 cells treated with TiO_2_ particles (0–5 μM) for 24 h exhibited a significant and dose-dependent increase in genotoxicity. Their findings are supported by Di Virgilio *et al*. [174] who also had similar results in CHO-K1 cells. Genotoxic effects were shown by MN frequencies, which significantly increased at 0.5 and 1 μg/ml of TiO_2_ NPs. SCE frequencies were higher for cells treated with 1–5 μg/ml of TiO_2_ NPs. Conversely, a chronic (60 days) study with CHO cells (0, 10, 20, and 40 μg/cm^2^) showed no cyto or genotoxic effects by TiO_2_ NPs (100% anatase, 25 nm) [175]. They stated that CHO cells adapted to chronic exposure and detoxified the excess ROS possibly through upregulation of superoxide dismutase (SOD).

A recent study found that TiO_2_ NPs (aeroxide P-25 99.5% 73–85% anatase, 14–17% rutile, and 2–13% amorphous) were cytotoxic and genotoxic to human skin fibroblast cell line in a dose dependent manner (10, 25, 50, 100, 250, 500, and 1000 μg/ml) using the test for γ-H2AX expression [176]. Another study specifically targeted the relationship between TiO_2_ NPs and the DNA damage response pathways regulated by ATM/Chk_2_ and ATR/Chk1 in human dermal fibroblasts [177]. Their results showed increased phosphorylation of H2AX, ATM, and Chk_2_ after exposure. In addition, TiO_2_ NPs inhibited the overall rate of DNA synthesis and frequency of replicon initiation events in DNA-combed fibres. Taken together, these results demonstrate that exposure to TiO_2_ NPs activates the ATM/Chk_2_ DNA damage response pathway.

The interaction of TiO_2_ NPs with liver DNA from ICR mice was systematically studied *in vivo* using ICP-MS, various spectral methods and gel electrophoresis [178]. The results showed that the liver weights of the mice treated with higher amounts of anatase TiO_2_ NPs were significantly increased. They stated that anatase TiO_2_ NPs could have accumulated in liver DNA by inserting itself into DNA base pairs or binding to DNA nucleotide that bound with three oxygen or nitrogen atoms and two phosphorous atoms of DNA with the Ti-O(N) and Ti-P bond lengths of 1.87 and 2.38 A, respectively, and could alter the conformation of DNA. The gel electrophoresis showed that higher dose of nano-anatase TiO_2_ NPs could cause liver DNA cleavage in mice.

However, as stated in the *in vivo* studies section, there are also studies that imply the opposite. A recent study found that TiO_2_ NPs (28 nm; 90/10: anatase/rutile) did not induce ROS production or increase the expression of γ-H2AX in A549 cells (0, 2.5, 5, 10, 15, 20, and 40 μg/ml; 24h) [179]. The transcription and protein expression levels of two Hsp members, Grp78 and Hsp70, were evaluated to ascertain their suitability as biomarkers of TiO_2_ NP-induced toxicity in the respiratory system [180]. Even though the presence of TiO_2_ NPs (25 nm) was confirmed in the cells *via* ultra-structural observations leading to cell death and induction of intracellular ROS generation, the transcription and protein expression levels of Hsp70 and Grp78 did not change at the same dose range (25–500 μg/ml) in A549 cells. They concluded that Hsp70 and Grp78 are not suitable biomarkers for evaluating the acute toxicological effects of TiO_2_ NPs in the respiratory system.

A recent study by Woodruff *et al*. [181] found that TiO_2_ NPs (10 nm anatase spheres; non-coated; 0-200 μg/ml; 24 h) were not genotoxic under the conditions of the Ames test and Comet assay in the thymidine kinase heterozygote (TK6) cell lines. There was no significant DNA damage or oxidative DNA damage observed. Warheit et al. [114] also reported negative results for an *in vitro* mammalian chromosome aberration test on Chinese hamster ovary cells (CHO) treated with TiO_2_ NPs (metaphase at 750, 1250, and 2500 μg/ml 4 h non-activated test condition; at 62.5, 125, 250 μg/ml, 4 h activated test condition, and at 25, 50, 100 μg/ml 20 h non-activated test condition). Linnainmaa *et al*. [182] reported similar negative results in cultured rat liver epithelial cells using the MN assay. Their results suggest that both TiO_2_ FPs and NPs (5, 10, and 20 μg/cm^2^) have no direct clastogenic potential. Fisichella *et al*. [183] concluded in their study that surface treated TiO_2_ NPs (100 μg/ml of STNP for 4, 24, and 72 h) with a rutile core (7±2 nm × 50±10 nm) are not harmful to Caco-2 cells. In TiO_2_ NP-induced inflammation, NF-κB is thought to be activated in response to pro-inflammatory cytokines. However, a recent study by Wilson *et*.*al*[184] showed that after 6 h incubation with P-25 (10, 50, and 250 μg/ml), NF-κB was not activated in A549 cells. They concluded that NF-κB DNA binding may not be the likely transcription pathway that leads to TiO_2_ NP-induced inflammation. TiO_2_ NPs also were found to have no effect on the regulation of plasminogen activator inhibitor-1 expression in endothelial cells [185]. TiO_2_ NPs did not cause an increase in pro-mitochondrial membrane potential (MMP)-2 and pro-MMP-9 gelatinolytic activities in conditioned media, there was no dose- and time-related decreases in tissue inhibitors of metalloproteinases 2 (TIMP-2) and no transcriptional change of TIMP-1 were observed in U937 cells [186].

In summary, many *in vivo* and *in vitro* studies were conducted to investigate the genotoxicity of TiO_2_ FPs and NPs, but results are conflicting. Some studies indicate that TiO_2_ NPs are genotoxic, whereas the others give negative results. Even though the rationale for these conflicting results is not clear, use of different cell types, exposure metrics, crystalline structure, particle dispersion and NP sizes may be an explanation. Most of the cell lines which show genotoxicity are cells associated with the respiratory system and the circulatory system. Overall, the studies indicating that TiO_2_ NPs are genotoxic outweigh the studies that state otherwise. Thus, TiO_2_ NPs can be treated as potential hazards. More studies are needed to determine the conditions in which TiO_2_ NPs genotoxicity occurs [32]. The possible mechanisms for TiO_2_ NP-induced genotoxicity involve DNA damage directly or indirectly *via* oxidative stress and/or inflammatory responses. Tables 1 and 2 give a summary of the genotoxicity studies mentioned in this paper.

**Table 1:** **Genotoxicity of TiO**_**2 **_**NPs *in vivo *studies**

**Reference No**.	**Crystalline structure**	**Exposure mode**	**Dose**	**Test type**	**Result**
[32]	P25 (75% anatase, 25% rutile)	Drink water (C57Bl/6Jmice)	60, 120, 300, and 600 μg/ml	Comet assay	(+)
				Micronuclei assay (PCE)	(+)
				γ-H2AX assay	(+)
				Immunostaining assay	(+)
				RT-PCR(TNF-α, IFN-γ, IL8)	(+)
				RT-PCR(TGF-β, IL-10, IL-4)	(−)
[115]	Nano-TiO^2^	intragastric administration	100,1000, and 5000 mg/kg	Micronuclei assay (PCE)	(−)
[142]	(20 nm), rutile (80 nm)	Inhalation (C57BL/6J mice)	200 μg/ml	ELISA assay (IL-1α, IL-1β, IL-6, and TNF)	(+)
[145]	Anatase	Inhalation (ICR mice)	0, 5,10, and 50 mg/kg	RT-PCR (IKK1, IKK2, NF-κB, NF-κBP52, NF-κBP65, TNF-α, and NIK)	(+)
				ELISA (IKK1, IKK2, NF-κB, NF-κBP52, NF-κBP65, TNF-α, and NIK)	(+)
[146]	Anatase	Intragastric administration (ICR mice)	0, 2.5, 5, 10 mg/kg	mRNA expression (NF-κB, TNF-α, Hsp70, IL-1α, MIF, INF-γ, TGF-β, CRP, CYP1A, IL-4,6,8,10,18)	(+)
				ELISA (NF-κB, TNF-α, Hsp70, IL-1α, MIF, INF-γ, TGF-β, CRP, CYP1A, IL-4,6,8,10,18)	(+)
[147]	Anatase	Intratracheal instillation (ICR mice)	0, 2.5, 5, 10 mg/kg	RT-PCR (NF-κB, IκB, TNF-α, IL-2, IL-4, IL-6, IL-8, IL-10, IL-18, IL-6, IL-1α, COX-2, HO-1, CYP1A1 and HSP-70)	(+)
				ELISA (NF-κB, IκB, TNF-α, IL-2, IL-4, IL-6, IL-8, IL-10, IL-18, IL-6, IL-1α, COX-2, HO-1, CYP1A1 and HSP-70)	(+)
[148]	Anatase, 100%	Intraperitoneal injection (ICR mice)	0, 5, 50, 150 mg/kg	RT-PCR (caspase-3, caspase-9, Bax, Bcl-2, and cytochrome c)	(+)
				ELISA (caspase-3, caspase-9, Bax, Bcl-2, and cytochrome c)	(+)
[149]	UV-Titanium (rutile, 17 nm)	Inhalation (C57BL/6BomTac mice)	42 mg/m^3^	DNA strand breaks	(−)
				DNA microarrays (Cyp26b1, Ttr, and Ugt3a2)	(+)
				RT-PCR (Cyp26b1, Ttr, and Ugt3a2)	(−)
[150]	P20 (coated with polyalcohol)	Inhalation (C57BL/6BomTac mice)	42.4 mg/m^3^	Gene Expression Analysis (Copine5, Saa1, and Saa3)	(−)
				DNA microarray	(−)
				PCR (cxcl-5, cxcl1, ccl2, ccl22, ccl7, ccr4, and TNF)	(+)
[151]	UV-Titanium (coated with polyalcohol, 20.6nm)	Inhalation (C57BL/6J mice)	42.4 mg/m^3^	Expanded simple tandem repeat (ESTR) assays	(−)
[152]	TiO^2^ (97%)	Inhalation (C57BL/6J mice)	0, 0.8, 7.2, 28.5 mg/m^3^	Micronuclei assay (PCE)	(−)
				Comet assay	(−)
[153]	P25 (15 nm, ultrafine)	Inhalation (Wistar rats)	10.4 mg/m^3^	DNA adduct	(+)
[178]	Anatase	Intraperitioneal injection (ICR mice)	0, 5, 10, 50, 100, and 150 mg/kg	DNA damage	(+)

(+) Positive; (−) Negative.

**Table 2:** **Genotoxicity of TiO**_**2 **_**NPs *in vivo *studies**

**Reference No**.	**Crystalline structure**	**Exposure mode**	**Concentration**	**Test type**	**Result**
[21]	Anatase	HepG2 cells	0, 1, 10, 100 and 250 μg/ml of TiO^2^ NPs	Comet assay	(+)
				Fpg-Comet	(+)
				Upregulated mRNA expression (p53)	(+)
	Rutile			Comet assay	(±)
				Fpg-Comet	(±)
				Upregulated mRNA expression (p53, mdm2, p21 and gadd45α)	(+)
[28]	Anatase, 99.7%	A431 cells	0.008 -80 μg/ml (10 times)	Comet assay	(+)
[30]	TiO^2^ NPs (99% pure)	Human lymph- oblastoid cells	130 μg/ml	Cytokinesis-block micronucleus (CBMN) assays	(+)
				HPRT mutation assay	(+)
			65 μg/ml	Comet assay	(+)
[93]	Anatase/Rutile, 80/20	NR8383 rat lung alveolar macrophages	0, 10, 20, 40, 80 μg/cm^2^	qRT-PCR (HO-1)	(+)
				Immunocytochemistry (NF-κB)	(+)
[114]	Rutile	CHO cells	0,25, 50, 100 μg/ml	Mammalian chromosome aberration test	(+)
[155]	Spherical (anatase & rutile)	A549 cells	100 μg/ml	Single strand breaks Comet assay	(+)
				HPLC-MS/MS	(+)
				8-oxodCuo	(+)
[156]	P30.6	WISH cells	0.625-20 μg/ml	Olive tail moment	(+)
				ROS generation	(+)
				Cell cycle arrest	(+)
[157]	Anatase (< 100 nm)	IMR90 cells BEAS-2B cells	0,2,5,10,50 μg/cm^2^	Olive tail moment	(−)
				DNA breaks	(−)
[158]	V_2_O_5_ treated TiO^2^ anatase	V79 cells	0,1,5,10,15,25 μg/cm^2^	Micronucleus test	(+)
				DNA damage	(+)
	Untreated anatase				
[159]	Spherical (anatase) Spicular (rutile)	THP-1 cells	0,20, 100, 500 μg/ml	ROS	(+)
[160]	Anatase, 99%	U937 cells	0.005-4 mg/ml	DNA fragmentation	(+)
[161]	Anatase/rutile, 80/20	RAW264.7 cells	0,1, 5, 10, 40 or 80 μg/cm^2^	DNA fragmentation	(+)
				ELISA (CDDE)	(−)
[162]	P25 (70-85% Anatase, 30-15% rutile)	Human perip- heral blood lymphocytes	0, 20, 50, 100 μg/ml	Flow cytometry of apoptosis	(+)
				Western blot (cleaved caspase-8, -3, Bid, and cleaved PARP)	(+)
				SiRNA transfection	(+)
[163]	Anatase	BEAS 2B cells	0, 5, 50, 100 μg/ml	PCR (Caspase 3 and PARP)	(+)
				SiRNA knockoutt Bid expression	(−)
				Western blot (bcl-2, bax, t-Bid, caspase 9, cytochrome C and p53)	(+)
[164]	P-25 (70–85% Anatase, 30–15% rutile)	Human perip- heral blood lymphocytes	0, 20, 50, 100 μg/ml	Comet assay	(+)
				Western-blot(p53, p63, phospho-p53, Chk1, phospho-Chk1, Chk2, phospho-Chk2, phospho-FKHR, phospho-FKHRL1)	(+)
[165]	P-25 (75% Anatase, 25% rutile)	HaCaT cells	200 μg/ml	Flow cytometry of apoptosis	(+)
				mRNA expression (Keratin 6)	(+)
[166]	TiO^2^ NPs	Human lymphocytes	0,2,4,6,8,10 mM	Comet assay	(+)
				DNA ladder assay	(+)
[167]	Rutile (>95%, <5% SiO^2^ coating) anatase (99.7%)	BEAS 2B cells	1-100 μg/cm^2^	Comet assay	(−)
				CBMN assay	(−)
				Comet assay	(+)
				CBMN assay	(+)
[168]	PEG-TiO^2^ NPs (P25 80% anatase, 20% rutile)	NCI-H292, HeLa and HepG2 cells	75 μg/ml	RT-PCR (CSF-2)	(−)
				RT-PCR (IL6, HMOX-1)	(+)
[169]	Anatase, anatase/rutile	Caco-2 cells	20, 80 μg/cm^2^	Fpg-comet assay	(+)
[170]	Anatase (10/20)	BEAS-2B cells	0, 5, 10 μg/ml	Micronucleus test	(+)
				Comet assay	(+)
[171]	P15	NIH 3T3 cells and human fibroblast HFW cells	10 μg/ml	Micronucleus assay	(+)
				ROS	(+)
			50 μg/ml	Colony forming assay	(+)
			0-100 μg/ml	Western blot (ERK, MEK)	(+)
[172]	Ultrafine (≤20 nm)	SHE cells	0,0.5, 1, 5, 10 μg/cm^2^	Micronucleus assay	(+)
				Kinetochore staining	(+)
				DNA fragmentation	(+)
				DNA ladder assay	(−)
[173]	P25 anatase	SHE cells	0,10, 25, 50 μg/cm^2^	Comet assay	(+)
[174]	TiO^2^ NPs	Chinese ham- ster ovary-K1 (CHO-K1) cells	0.5, 1 μg/ml	Sister chromatid exchange (SCE)	(+)
				Micronucleus assay	(+)
[175]	Anatase (100% <25 nm)	Chinese ham- ster ovary-K1 (CHO-K1) cells	0, 10, 20, 40 μg/ml	Comet assay	(−)
				Gene mutation assay (Hprt)	(−)
[176]	P25(99.5% purity, 73–85% anatase/14–17% rutile and 2–13% amorphous)	Human neonatal foreskin fibroblast cells	0, 10, 25, 50, 100, 250, 500, 1000 μg/ml	DNA damage	(+)
				Immunofluorescent (γ-H2AX)	(+)
[177]	Anatase (15 nm, 100%)	Human dermal fibroblasts	0, 1, 3, 10 μg/ml	DNA damage (ATM/Chk2)	(+)
[179]	P28 (anatase 90%, rutile 10%)	A549 cells	0, 5, 15 μg/ml	ROS	(−)
				DNA double strand breaks (γ-H2AX)	(−)
[180]	P25	A549 cells	25-500 μg/ml	mRNA expression (Grp78	(−)
				and Hsp70)	
				Western blot (Grp78	(−)
				and Hsp70)	
[181]	TiO^2^ NPs	TA-100 cells	200 μg/ml	Comet assay	(−)
[182]	P25, UV-titan M60	Rat liver epithelial cells	0, 5, 10, 20 μg/ml	Micronucleus assay	(−)
[183]	Surface treated rutile TiO^2^	Caco-2 cells	100 μg/ml	Gene expression analysis	(−)

(+) positive; (−) negative; (±) uncertain.

### Reproductive and developmental toxicity

Although experimental evidence shows that absorbed TiO_2_ particles may be able to move across the placenta into fetal tissue, it has not yet been established whether human exposure to TiO_2_ particles causes reproductive and developmental toxicities. Exposure of other species, such as zebra fish [187] and abalone embryo [113], to TiO_2_ particles have shown that it can impair reproduction, inhibit hatching, and cause malformations. However, in the case of zebra fish some disagree [188]. In mammals, limited animal data are available to define the developmental or reproductive toxicity of TiO_2_ NPs. With respect to *in vivo* studies, Takeda *et al*. [189] demonstrated that prenatal subcutaneous exposure of mice to TiO_2_ NPs (25 and 70 nm; 16 mg/kg) at day 3, 7, 10, and 14 can damage the genital and cranial nerve systems in the offspring. In this study, TiO_2_ NPs identified by energy-dispersive X-ray spectroscopy were found in the testes and brain of exposed 6-week-old male mice, which indicated that TiO_2_ NPs may penetrate both blood-testis and BBB. Shimizu *et al*. [88] reported that, in the brain tissue of male fetuses (embryonic day 16) and pups (postnatal days 2, 7, 14, and 21), subcutaneous injection of pregnant mice (100 μl TiO_2_ NPs suspended at 1 μg/μl) altered expression of genes associated with brain development, cell death, response to oxidative stress, and mitochondrial activity in the brain during the perinatal period [88]. Even though subcutaneous exposures may not be realistic, this study does show that the fetal nervous system is specifically sensitive to maternal TiO_2_ NPs exposure during pregnancy. Moderate alterations in neurobehavior were also noted by Hougaard *et al*. [90] in mated C57BL/6BomTac mice exposed (1 h/day) through inhalation (42 mg/m^3^) to surface coated TiO_2_ NPs (UV-Titan; 97 nm) on GD 8–18. Yamashita *et al*. [190] reported that silica and TiO_2_ NPs with diameters of 70 and 35 nm, respectively, can cause pregnancy complications when injected intravenously into pregnant mice. The TiO_2_ NPs were found in the placenta, fetal liver and fetal brain. Mice treated with TiO_2_ NPs had smaller uteri and smaller fetuses than untreated controls.

Komatsu *et al*. [191] investigated the effects of TiO_2_ NPs on mouse testis Leydig cells *in vitro* and found TiO_2_ NPs were more cytotoxic to Leydig cells than diesel exhaust and carbon black NPs. TiO_2_ NPs were taken up by Leydig cells, and affected viability, proliferation and gene expression.

In summary, limited *in vivo* and *in vitro* studies suggest that TiO_2_ NPs exposures may exert certain reproductive and developmental toxicities. Further studies are needed to clarify the mechanisms underlying these toxicity results.

### Carcinogenicity

The mechanisms of metal-induced carcinogenesis are not well understood. Both genetic and non-genetic factors elicited by TiO_2_ NPs in cells may predispose to carcinogenicity [176].

#### Experimental studies

Animal experimental studies show that high concentrations of TiO_2_ FPs (<2.5 um; 250 mg/m^3^; 2 yrs) and TiO_2_ NPs (<100 nm; 10 mg/m^3^; 2 yrs) can cause respiratory tract cancer in exposed rats [43,192]. Chronic lung inhalation studies have shown that TiO_2_ NPs can cause bronchoalveolar adenomas and cystic keratinizing squamous cell carcinomas at high doses [9] and alveolar/bronchiolar adenoma [193]. Heinrich *et al*. [194] investigated the carcinogenicity of TiO_2_ NPs (15–40 nm) and found TiO_2_ NPs were tumorigenic in rats at a concentration of approximately 10 mg/m^3^ for 2 years, followed by a 6-month holding period. TiO_2_ NPs seem to have more carcinogenic potential in the rat than TiO_2_ FPs on an equal mass dose basis. This difference in carcinogenic potency suggests the need to develop separate risk estimates for TiO_2_ FPs and NPs exposures, and to develop separate recommendations for occupational exposures to each size range [43].

To assess the health risks of occupational exposure to TiO_2_ NPs, Kuempel *et al*. [76] extrapolated rodent data to humans using a lung dosimetry model. The rat-based estimates of the working lifetime airborne concentrations of TiO_2_ NPs associated with 0.1% excess risk of lung cancer were approximately 0.07 to 0.3 mg/m^3^. Using a similar model, Dankovic *et al*. [43] extrapolated rat threshold estimates to lifetime human occupational exposures and found a range of estimated occupational exposure levels of 0.8-5.8 mg/m^3^ for TiO_2_ FPs, and 0.09–0.66 mg/m^3^ for TiO_2_ NPs. Such risk analyses formed the basis for development of RELs of 0.3 and 2.4 mg/m^3^ for TiO_2_ NPs and FPs, respectively [50]. Due to the lack of human epidemiological information, these extrapolation models using animal experimental data are still useful in the prediction for risk assessment of occupational exposure to TiO_2_ NPs.

In an intratracheal instillation study female rats were administered TiO_2_ hydrophilic or anatase NPs (21–25 nm; 1/week for 30 weeks) of different doses [195]. The incidence of lung tumors (52–69.6%, adenomas/carcinomas and squamous cell epitheliomas/carcinomas combined) in rats receiving TiO_2_ hydrophilic or anatase NPs was significantly higher than controls (0%). Anatase NPs also significantly induced higher incidence of lung tumors (30–63.6%). The incidence of benign and malignant lung tumors in the TiO_2_ hydrophilic NPs groups (6.7%) was not significant. The incidences of cystic keratinizing epitheliomas (11.7%) and squamous cell carcinomas (4.8%) were significantly greater than the control group (0.5%) in another inhalation study with female rats treated with TiO_2_ particles (particle size not stated; 11.3 mg/m^3^; 24 months, followed by 6 months observation) [196]. Bernard *et al*. [197] conducted toxicological and carcinogenesis studies of dietary TiO_2_-coated mica in rats fed diets containing 0, 1.0, 2.0, or 5.0% TiO_2_-coated mica for up to 130 weeks. They found no evidence that TiO_2_-coated mica produced either toxicological or carcinogenic effects at dietary concentrations as high as 5.0%.

In regards to skin cancers, a two-stage skin model was used by Xu *et al*. [71] to examine the promoting/carcinogenic effect TiO_2_ NPs (rutile, 20 nm). C-Ha-ras proto-oncogene transgenic (Hras128) rats, which are sensitive to skin carcinogenesis and their wild-type siblings were exposed to UV-B radiation on shaved back skin twice weekly for 10 weeks. The shaved area was then painted with a 100 mg/ml TiO_2_ NP suspension twice weekly until sacrifice. The tumor incidence was not different from the UV-B controls. They suggested that TiO_2_ NPs does not cause skin carcinogenesis, which may be due to its inability to penetrate through the epidermis and reach underlying skin structures. The same conclusion was reached by Sagawa *et al*. [70] who studied the promoting effect of silicone coated TiO_2_ NPs (35 nm; 5 times a week for 8 and 40 weeks; 0, 10, or 20 mg) suspended in silicone oil and non-coated TiO_2_ NPs (20 nm; twice a week for 28 or 40 weeks; 0, 50, or 100 mg) suspended in Pentalan 408 on a two-stage skin chemical carcinogenesis model. Analysis of skin indicated that silicone coated TiO_2_ NPs and non-coated TiO_2_ NPs did not penetrate though either healthy or damaged skin. Newman *et al*. [65] also suggested that TiO_2_ NPs are not carcinogenic to the skin because they do not penetrate the intact dermal tissue. However, the authors emphasized that further studies for the safety evaluation of the TiO_2_ NPs in sunscreens must be done to simulate real-world conditions particularly in sunburned skin and under UV exposure.

Pulmonary studies support the carcinogenicity of TiO_2_ NPs in intratracheal and inhalation studies. However, exposure modes such as intragastric or dermal exposure do not indicate that TiO_2_ NPs are carcinogenic.

#### Epidemiological studies

Epidemiological studies on workers exposed to TiO_2_ particles, thus far, have not been able to detect an association between the occupational exposure and an increased risk for lung cancer. Furthermore, most studies were not designed to investigate the relationship between TiO_2_ particle size and lung cancer risk, which represents an important question for assessing the potential occupational carcinogenicity of TiO_2_ NPs [32]. The results from the epidemiological studies that have been conducted (no particle size defined) show that there are no significant associations between TiO_2_ exposure and risk of lung cancer [198,199], elevated standardized mortality ratio (SMR) for cancer [200], and reduction in ventilatory capacity [119]. As a whole, these epidemiological studies imply that occupational exposures to TiO_2_ FPs (or total dust) are not associated with increasing risk of cancers. Unfortunately, epidemiological studies of adverse health effects induced by TiO_2_ NPs alone are lacking. The relatively short history in production and use seems to be the main reason for the lack of human epidemiological studies for TiO_2_ NPs. Furthermore, it is difficult to make reliable hazard assessments of manufactured NPs, because the NPs may form large agglomerates in both *in vitro* and *in vivo* studies [51]. Experimental evidence supports that TiO_2_ NP agglomeration increases when the surface area decreases at a constant pH and that the isoelectric point for TiO_2_ depends on the particle size [201]. The interaction of this increased surface area with the biological environment induces oxidative stress [8]. It is worth noting that although TiO_2_ NPs are prone to forming agglomerates of >100 nm in suspension, these agglomerates are not stable and may dissociate in bodily fluids and tissues. However, the extent of such dissociation has not yet been determined. To evaluate the health effects of TiO_2_ NPs on workers, further well designed epidemiological studies are needed. The animal studies that have been conducted (those mentioned in this paper) indicate potential risk factors that could be assessed in occupational settings. Examples of potential risk factors are the underlying health of the workers and co-exposures.

In summary, available epidemiological studies as well as *in vivo* animal experimental data concerning the carcinogenic effects of TiO_2_ particles are outlined above. Epidemiological studies on workers exposed to TiO_2_ FPs failed to detect an association between the occupational exposure and an increased risk for cancer. Available data from human studies on TiO_2_ NPs exposures alone are still lacking. Carcinogenicity studies in animals indicate that TiO_2_ NPs can produce tumors when exposed through inhalation or intratracheal instillation and are more carcinogenic on an equal mass basis than TiO_2_ FPs. The tumors preferentially include adenomas and squamous cell carcinomas. Based on the studies outlined above, TiO_2_ NPs were evaluated by World Health Organization (WHO)/IARC as a Group 2B compound [202]. An overview of currently available carcinogenicity data on TiO_2_ NPs from experimental animals raises serious questions as to their health and environmental safety. Therefore, all commercially available TiO_2_ NPs should be assessed and their production and application should be managed appropriately. At this stage, risk characterization of TiO_2_ NPs is hampered by incomplete or lack of data on human exposure and dose- response analysis.

### Molecular mechanisms of carcinogenesis

Many studies have shown that TiO_2_ FPs and NPs induce cytotoxicity and genotoxicity in various cultured cell lines as well as tumorigenesis in animal models [171]. As stated above, DNA strand breaks, mutations, chromosomal damage and cell transformation have been observed in some *in vitro* or *in vivo* studies. However, the exact mechanisms of TiO_2_ NP-induced carcinogenesis are not clear. Recent evidence indicates that ROS formation, induction of inflammation and alterations in cell signal transduction induced by TiO_2_ NPs may play an important role in the etiology of their carcinogenesis. Elevated levels of ROS and down regulation of ROS scavengers and antioxidant enzymes are associated with various cancers [203]. ROS consist of a group of partially reduced forms of molecular oxygen, such as hydroxyl radical (•OH), superoxide anion (O_2_^−•^), singlet oxygen (^1^O_2_), hydrogen peroxide (H_2_O_2_), lipid peroxides, and hypochlorous acid (HClO) [204]. Accumulation of ROS may be accompanied by the production of reactive nitrogen species [205], such as the highly reactive peroxynitrite anion, a strong oxidant formed by the reaction of O_2_^−•^ and NO•. The cumulative production of ROS through either endogenous or exogenous insults is termed oxidative stress. Oxidative stress induces a cellular redox imbalance found in various cancer cells. ROS could induce non-selective DNA damage, which may result in genetic changes in active genes. Oxidative damage to cellular DNA can lead to mutations. The mutations in DNA may be involved in the initiation of various cancers. Therefore, oxidative stress induced by ROS generation may play an important role in the initiation and progression of multistage carcinogenesis of TiO_2_ NPs. The generation of ROS and induction of inflammation leading to alterations of signaling components due to TiO_2_ NP exposures are reviewed in this paper.

#### Generation of ROS

It has been hypothesized that insoluble particle accumulation in the animal lungs is mechanistically linked to the development of lung tumors [43]. Accumulation of TiO_2_ NPs in the lung leads to chronic inflammation, which may further lead to the formation of ROS and epithelial proliferation, and eventually lead to mutations and tumor formation. Some of the studies cited previously have reported the involvement of free radicals in DNA damage [28,157], ROS-induced activation of p53-mediated DNA damage check point signals [164], increased intracellular ROS leading to increased G_2_M cell cycle arrest or delay [156,171], cell-derived oxidants involved in induction of mutagenesis [144], and increased extracellular ROS coupled with HO-1 and NOS mRNA expression and TNF-α release [93]. Others have linked TiO_2_ NPs (Wistar rats; 1, 5, 10, 25, and 50 μg/ml; <25 nm; 1 h) to ROS generation as a result of mitochondrial dysfunction in lung tissues [206]. Jaeger *et al*. [207] investigated whether ROS-induced mitochondrial DNA damage is the mode of action by which TiO_2_ NPs (≤20 nm) induce cytotoxic and genotoxic effects in human HaCaT keratinocytes *in vitro*. They demonstrated the induction of the mitochondrial "common deletion" in HaCaT cells following exposure to TiO_2_ NPs. They proposed a ROS-mediated (increased 16.7 fold of control; 4 h; 5 and 50 μg/ml) cytotoxic and genotoxic potential for TiO_2_ NPs. They also showed that TiO_2_ enters the cell by endocytosis. Another study with HaCaT cells also showed that TiO_2_ NPs (anatase, rutile and anatase/rutile; 4, 10, 21, 25, and 60 nm; 200 μg/ml) could induce the generation of ROS and damage the cells under ultraviolet-A (UVA) irradiation [20] . The induced ROS resulted in oxidative stress in the HaCat cells, reducing SOD and increasing MDA levels. The cell viability was also decreased in a dose dependent manner. Reduction of cell viability and increased ROS generation by TiO_2_ NPs under UVA irradiation was also noted by Sanders *et al*. [208]. Similar effects were seen in a recent study by Yin *et al*. [209] on HaCat cell cultures. Yoo *et al*. [210] also stated that sub-100 nm sized TiO_2_ treatment under UVA irradiation induces apoptotic cell death through ROS-mediated up regulation of the death receptor, Fas, and activation of the pre-apoptotic protein, Bax.

In addition to these, a long term study (intratracheal administration; 2.5, 5, and 10 mg/kg) found that TiO_2_ NPs (5–6 nm) caused oxidative damage in lungs and enhanced expression of (Nrf_2_), HO-1 and glutamate-cystine ligase catalytic subunit (GCLC) on days 15 and 75 which began to decrease on day 90 [211]. They suggested that induction of Nrf_2_ expression was an adaptive intracellular response to TiO_2_ NP-induced oxidative stress in mouse lung. Another study on PC12 cells found that TiO_2_ NPs (1, 10, 50, and 100 μg/ml; 6, 12, 24, and 48 h) caused generation of ROS in a dose and time-dependent manner, leading to apoptosis [212]. ROS-mediated oxidative stress, the activation of p53, Bax, and caspase-3, as well as oxidative DNA damage were found to be involved in the mechanistic pathways of apoptosis induced by TiO_2_ NPs (anatase; 25 nm; 50, 100, and 200 μg/ml; 24, 48, and 72 h) in human embryonic kidney (HEK) 293 cells [213]. These increases followed a dose-dependent pattern. Wu *et al*. [214] also showed the involvement of p53 and JNK activation in G2/M cell cycle arrest and apoptosis induced by anatase TiO_2_ NPs (20 nm; 25, 50, 100, and 200 μg/ml) in the neuronal cell, PC12. A study on dendritic cells also found that TiO_2_ NPs enhanced ROS production [215]. Wang *et al*. [216] found that TiO_2_ NPs (intragastric exposure; 30 consecutive days) exerted toxicity on the mouse spleen through oxidative stress with significant increases in ROS. This subsequently led to strong LPO and the significant expression of HO-1 *via* the p38-Nrf-2 signaling pathway.

Uchino *et al*. [217] showed that the crystal size of TiO_2_ NPs (anatase; 30 nm; 50μg/ml) had large influence on •OH generation, but the optimum size for the •OH generation was different between both crystalline forms (anatase and rutile). A significant relationship was observed between cytotoxicity and •OH generation in CHO cells. TiO_2_ NPs (rutile; 40–70 nm, minor axis; 200–300 nm, major axis; 40–55 g/100g) were also shown to have the potential to convert benign tumor cells into malignant ones through the generation of ROS in the target cells [218]. However, apart from all of these studies implicating TiO_2_ NP-generated ROS in cellular and molecular effects, a recent article by Toyooka *et al*. [219] demonstrated that TiO_2_ NPs could cause DNA damage without generating ROS. Their study examined the genotoxicity of two different sizes of TiO_2_ NPs in the A549 cells based on the phosphorylation of γ-H2AX. Flow cytometric analysis showed that the generation of γ-H2AX by TiO_2_ NPs was independent of cell cycle phases, and cells which incorporated larger amounts of TiO_2_ particles had more significant γ-H2AX.

#### Induction of inflammation

Most of the studies previously mentioned in other sections have also reported inflammatory effects due to TiO_2_ NPs exposure. The details of the molecular events involving inflammation for some of these studies will be discussed here. Cytokines are components of the immune system that are involved in these molecular events, either as agonists or antagonists of inflammation. TiO_2_ NPs (anatase; 20 nm; rutile, 80 nm; 7.5-30 mg/kg) signaled the interleukin 1 (IL-1α) family of cytokines in a mouse lung model [142]. The signaling of IL-1R by TiO_2_ NPs is similar to that of asbestos. Others showed that IL-1β production was depended on active cathepsin B and ROS production independent of the characteristics of TiO_2_[159]. Another study showed increased expression of IL-1β, IL-2, IL-4, IL-6, IL-10, and IL-18, in nephritic inflammation caused by TiO_2_ NPs intragastrically administered to mice (5–6 nm; 2.5, 5, and 10mg/kg; everyday for 90 days) [146]. In addition, TiO_2_ NPs activated NF-κB, leading to increased expression of TNF-α, MMIF, cross-reaction protein, TGF-β, interferon-γ, and CYP1A1, and decreased Hsp70 expression. Moon *et al*. [220] showed that the levels of pro-inflammatory mediators, such as IL-1β, TNF-α, and macrophage inflammatory protein (MIP)-2, in BALF and mRNA expression of TNF-α and IL-1β in lung tissue were elevated post-exposure in mice (intraperitoneal; 40 mg/kg BW). TiO_2_ NP exposure increased neutrophil influx, protein levels in BALF, and ROS activity of BAL cells 4 h after exposure. In addition, TiO_2_ NP exposure resulted in significant activation of inflammatory signaling molecules, such as c-Src and p38 mitogen-activated protein kinase (MAPK), in lung tissue and alveolar macrophages. Activation of the NF-κB pathway in pulmonary tissue was also noted. Kan *et al*. [143] showed that TiO_2_ NPs increased phosphorylation of p38 and troponin 1 in cardiac muscle. It can be seen here that the induction of inflammation by TiO_2_ NPs involves a host of other molecular components and events including the signaling of cytokines. Additional evidence for this is shown by the following references [145,154,160,162,180].

Recent research indicates that TiO_2_ particle-induced alterations in signal transduction may also play an important role in the etiology of cancer. Goncalves *et al*. [221] investigated the *in vitro* effects of TiO_2_ NPs on human neutrophils. Kinetic experiments revealed no cell necrosis after a 24 h treatment with TiO_2_ NPs (0–100 μg/ml). However, TiO_2_ NPs markedly and rapidly induced tyrosine phosphorylation events, including phosphorylation of two key enzymes, p38 MAPK and extracellular signal-regulated kinases-1/2 (Erk-1/2). Supernatants from induced neutrophils were collected after 24 h and tested for the presence of 36 different analytes (cytokines, chemokines) using an antibody array assay. TiO_2_ NP treatment increased production of 13 (36%) analytes, including IL-8, which exhibited the greatest increase (approximately 16 fold increase compared to control). These results indicate TiO_2_ NPs exert important neutrophil agonistic properties *in vitro* which represents one of the characteristics of carcinogens. Chen *et al*. [118] pointed out that a mixture of anatase and rutile TiO_2_ NPs (<100 nm, anatase/rutile 99.5% trace metal basis; 0–0.75 mg/ml) induced histamine secretion in mast cells (RBL-_2_H_3_ cells). Mast cell exposure to TiO_2_ NPs activated membrane L-type Ca^2+^ channels, induce ROS production and stimulate PLC activity. Influx of extracellular Ca^2+^ raises [Ca^2+^] _i_, and when coupled with the IP_3_-IP_3_ receptor pathway can trigger the release of ER resident Ca^2+^ and subsequent histamine secretion. They stated that TiO_2_ NPs directly trigger inflammatory mediators, thus bypassing traditional immuno-stimulation by allergens. These results suggest that mast cell degranulation of histamine may be significantly augmented and intensified in TiO_2_ NP exposed tissues with or without IgE antibody-based sensitization.

In conclusion, research evidence seems to be sufficient to conclude that both TiO_2_ FPs and NPs generate ROS as demonstrated by both *in vivo* and *in vitro* studies. ROS-induced signaling and activation of the IL family of cytokines, Bax, caspases 3 and 9, NF-κB, and p53, as well as phosphorylation of p38 and G_2_M phase cell cycle arrest seem to be common findings. In regards to induction of inflammation leading to the production of ROS, inflammatory cytokines seem to play an influencing role. Furthermore, experimental data suggest that ROS generation and oxidative stress may be important in TiO_2_ NP-induced genotoxicity and carcinogenicity. The exact mechanisms of TiO_2_ NP induced carcinogenesis are not clear. Limited data show that ROS, oxidative stress, as well as, cell signaling alterations of carcinogenic genes may all play significant roles in the carcinogenicity of TiO_2_ NPs at relatively high doses. Further studies are needed employing lower, occupationally relevant doses, which avoid the confounding influence of possible overload.

### Summary

Conventionally, TiO_2_ FPs have been considered as a low toxicity material. TiO_2_ NPs possess different physicochemical properties compared to TiO_2_ FPs, which would be expected to alter their biological properties. A full risk assessment for various routes of exposure to TiO_2_ NPs requires further data. Apart from the NIOSH recommended REL, to date, no occupational or environmental exposure limits for TiO_2_ NPs have been set by any other regulatory agency. Current understanding on their toxicity largely depends on a limited number of experimental animal or cell culture studies, where extrapolation to human exposures is required. Epidemiological studies thus far have not been able to detect an association between the occupational exposure to TiO_2_ particles and an increased risk for cancer. The physicochemical properties of TiO_2_ NPs may strongly influence their bioavailability and toxicity. Majority of data imply that TiO_2_ anatase NPs are cytotoxic or genotoxic. However, this conclusion was based on studies using TiO_2_ anatase NPs only. Under conditions of occupational exposure, inhalation of TiO_2_ NPs is normally the principal route for entry into the human body. Pulmonary inflammatory responses and lung cancers are the most important adverse effect observed in experimental animals due to TiO_2_ NP exposures. When only using realistic doses are considered, as in the case of some inhalation studies, inflammatory responses are still a prominent effect seen. TiO_2_ NPs can be absorbed through the lung or GIT into systematic circulation and then distributed in different organs such as liver, kidneys, spleen, or even brain causing localized effects. However, the rate of such translocation is currently uncertain. Some evidence has shown that TiO_2_ NPs cannot penetrate the intact skin into the human body. TiO_2_ NPs may have the potential to penetrate the blood–brain, blood-testis and blood-placenta barriers. However, the rate of translocation appears low and evidence is lacking which link systemic responses to translocation of particles to systemic sites. Many studies have been conducted *in vitro* and *in vivo* to investigate the genotoxicity of TiO_2_ NPs but the results are conflicting and doses employed were high. Certain reproductive and developmental toxicities in experimental animals or cell cultures have been observed in a few *in vivo* and *in vitro* studies. Whether human exposure to TiO_2_ NPs causes reproductive and developmental toxicities is unclear. Animal studies imply that accumulation of TiO_2_ NPs in organs or tissues may take place after continuous exposure. Responses to accumulation of TiO_2_ NPs in systemic organs need to be evaluated in further studies. In addition, TiO_2_ NP-induced generation of ROS and alterations in cell signal transduction pathways may play an important role in the etiology of carcinogenesis of TiO_2_ NPs at relatively high doses. However, these studies should be repeated at doses relevant to normal occupational or environmental exposure conditions where particle overload is not an issue. Despite this, the results currently available imply that TiO_2_ NPs exhibit greater toxicity than TiO_2_ FPs. These data should not be ignored, and development of prevention strategies to protect worker's health appears to be a prudent course of action.

In summary, although TiO_2_ NPs have been studied extensively in recent years, there is still much remaining to be elucidated concerning their possible health effects to support risk assessment and management.

First, to assure worker and consumer safety, it is urgently important to conduct exposure hazard assessment, which would allow the development of a framework enabling risk management for all commercial TiO_2_ NPs. This also includes bio-safety evaluation of TiO_2_ nanoparticulate carriers for drug delivery application.

Second, all future studies on TiO_2_ NPs should characterize the physicochemical properties of the NPs, such as size distribution, crystalline structure, surface area, surface coating, etc., as delivered to the biological system. This will allow for better comparison of data from different studies and assist in determination of appropriate dosimetry.

Third, long-term animal studies comparing the toxicity and carcinogenicity of TiO_2_ FPs and NPs are especially needed. The focus of these studies must be aimed at both occupational and consumer relevant doses and routes of exposure.

Fourth, detailed toxicokinetics studies that include absorption, distribution, metabolism, accumulation, and excretion of TiO_2_ NPs through different exposure routes into the human body are indispensable. In addition, future studies should focus on evaluating systemic responses distinct from the organ of exposure and biomarkers reflecting TiO_2_ NP exposure and toxic effects.

Finally, the molecular mechanisms by which TiO2 NPs may cause cancer are unclear. Limited data show that ROS generation and signal alterations of certain cancer-related genes may be involved in the carcinogenicity of TiO_2_ NPs. Therefore, further investigation is needed to elucidate the molecular mechanisms of carcinogenicity for TiO_2_ NPs.

## Abbreviations

(3-D-HB): 3-D-hydroxybutyrate; (DOPAC): 3,4-dihydroxyphenylacetic acid; (5-HT): 5-hydroxytryptamine; (5-HIAA): 5-hydroxyindole acetic acid; (AOO): Acetone-Olive Oil; (AHR): Airway hyper-reactivity; (ALT): Alanine aminotransferase; (ACGIH): American Conference of Governmental Industrial Hygienists; (AST): Aspartate aminotransferase; (BER): Base excision repair; (BBB): Blood brain-barriers; (BUN): Blood urea nitrogen; (BW): Body weight; (BALF): Bronchoalveolar lavage fluid; (BEAS-2B): Bronchial Epithelial; (CHO-K1): Chinese hamster ovary-K1; (Hras128): C-Ha-ras proto-oncogene transgenic; (CK): Creatine kinase; (CBMN): Cytokinesis block micronucleus; (DA): Dopamine; (ELISA): Enzyme-linked immunosorbent assay; (ESTR): Expanded simple tandem repeat; (FcγRII): Fcγ receptor II; (FP): Fine particle; (FPs): Fine particles; (FDA): Food and Drug Administration; (GIT): Gastrointestinal tract; (GD): Gestation days; (GCLC): Glutamate-cystine Ligase Catalytic Subunit; (GST): Glutathione S-transferase; (Hsp70): Heat shock protein 70; (HO-1): Hemeoxygenase-1; (HDL-C): High density lipoprotein cholesterol; (HVA): Homovanillic; (HEK): Human embryonic kidney; (1H-NMR): Hydrogen-1 nuclear magnetic resonance spectroscopy; (HClO): Hypochlorous acid; (H2O2): Hydrogen peroxide; (•OH): Hydroxyl radical; (HPRT): Hypoxanthine phosphoribosyltransferase; (IKK-α IKK-β): IκB kinases; (IL): Interleukin; (IARC): International Agency for Research on Cancer; (LDH): Lactate dehydrogenase; (LPO): Lipid peroxidation; (LEV): Local exhaust ventilation; (LDL-C): Low density lipoprotein cholesterin; (MIP): Macrophage inflammatory protein; (MMIF): Macrophage migration inhibitory factor; (MDA): Malodialdehyde; (LD50): Median lethal dose; (LC50): Median lethal concentration; (MN): Micronucleus; (MMP): Mitochondrial membrane potential; (MAPK): Mitogen-activated Protein Kinase; (NAC): N-acetylcysteine; (NP): Nanoparticle; (NPs): Nanoparticles; (NIOSH): National Institute for Occupational Safety and Health; (NEDO): New Energy and Industrial Technology Development Organization; (NIK): NF-κB-inducible kinase; (NO): Nitric oxide; (NOS): Nitric oxide synthase; (NOAEL): No Observed Adverse Effect Level; (NE): Norepinephrine; (NF-κB): Nuclear factor kappa-light-chain-enhancer of activated B cells; (NER): Nucleotide excision repair; (OSHA): Occupational Safety & Health Administration; (OTM): Olive tail moment; (OECD): Organization for Economic Co-operation and Development; (OVA): Ovalbumin; (PM): Particulate matter; (PEL): Permissible exposure limit; (PAG): Phenylacetylglycine; (PDT): Photodynamic therapy; (PCE): Polychromatic erythrocyte; (PEG): Polyethylene glycol; (ROS): Reactive oxygen species; (REL): Recommended exposure limit; (RDI): Relative deposition index; (RP-HPLC): Reversed-phase high performance liquid chromatography; (RT-PCR): Real-time quantitative PCR; (SEM): Scanning electron microscopy; (Saa1): Serum amyloid A-1; (Saa3): Serum amyloid A-3; (SGOT): Serum glutamic oxaloacetic transaminase; (SGOT): Serum glutamic pyruvic transaminase; (1O2): Singlet oxygen; (SiO2): Silicon dioxide; (SCE): Sister chromatid exchange; (SMR): Standardized mortality ratio; (SC): Stratum corneum; (O2−•): Superoxide anion; (SOD): Superoxide dismutase; (TEM-EDX): TEM-coupled Energy Dispersive X-ray; (TOF-SIMS: Time of Flight Secondary Ion Mass Spectrometry; (TWA): Time weighted average; (TIMP-2): Tissue inhibitors of metalloproteinases 2; (Ti): Titanium; (TiO2): Titanium dioxide; (TLV): Threshold limit value; (TLR2): Toll-like receptor-2; (TDI): Toluene diisocyanate; (TC): Total cholesterol; (TGF- β): Transforming growth factor-β; (TEM): Transmission electron microscopy; (TG): Triglyceride; (TMAO): Trimethylamine-N-oxide; (TNF-α): Tumor necrosis factor-α; (UVA): Ultraviolet-A; (UVB): Ultraviolet-B; (V2O5): Vanadium pentoxide; (WHO): World Health Organization

## Competing interests

The authors declare that they have no competing interests.

## Authors’ contributions

HS and RM were involved in writing the manuscript, JZ and VC helped to organize and proof read the final manuscript. All authors read and approved the final manuscript.

## Authors’ information

Miss Hongbo Shi and Ruth Magaye are graduate students at Ningbo University, China. Their current research focus is on nanotoxicology of nanomaterials. As authors or co-authors they have collectively published 7 scientific manuscripts.

Vincent Castranova, Ph.D., is the Chief of the Pathology and Physiology Research Branch in the Health Effects Laboratory Division of the National Institute for Occupational Safety and Health, Morgantown, West Virginia. Dr. Castranova’s research interests have been concentrated in pulmonary toxicology and occupational lung disease. He has been coordinator of the Nanotoxicology Program in NIOSH since its inception in 2005. He has been a co-editor of four books and has co-authored over 540 manuscripts and book chapters.

Dr. Jinshun Zhao works is a professor and director of the Public Health Department of Medical School of Ningbo University, Ningbo, Zhejiang, China. Dr. Jinshun Zhao’s research interests have been concentrated in occupational and environmental disease, toxicology and molecular mechanisms of chemical and metal-induced carcinogenesis. His current research is focused on the toxicology of nanomaterials. As an author or a co-author, he has published over 100 scientific manuscripts.
